# Practices and Applications of Convolutional Neural Network-Based Computer Vision Systems in Animal Farming: A Review

**DOI:** 10.3390/s21041492

**Published:** 2021-02-21

**Authors:** Guoming Li, Yanbo Huang, Zhiqian Chen, Gary D. Chesser, Joseph L. Purswell, John Linhoss, Yang Zhao

**Affiliations:** 1 Department of Agricultural and Biological Engineering, Mississippi State University, Starkville, MS 39762, USA; gl565@msstate.edu (G.L.); john.linhoss@msstate.edu (J.L.); 2 Agricultural Research Service, Genetics and Sustainable Agriculture Unit, United States Department of Agriculture, Starkville, MS 39762, USA; yanbo.huang@usda.gov; 3 Department of Computer Science and Engineering, Mississippi State University, Starkville, MS 39762, USA; zchen@cse.msstate.edu; 4 Agricultural Research Service, Poultry Research Unit, United States Department of Agriculture, Starkville, MS 39762, USA; joseph.purswell@usda.gov; 5 Department of Animal Science, The University of Tennessee, Knoxville, TN 37996, USA

**Keywords:** deep learning, convolutional neural network, computer vision system, animal farming

## Abstract

Convolutional neural network (CNN)-based computer vision systems have been increasingly applied in animal farming to improve animal management, but current knowledge, practices, limitations, and solutions of the applications remain to be expanded and explored. The objective of this study is to systematically review applications of CNN-based computer vision systems on animal farming in terms of the five deep learning computer vision tasks: image classification, object detection, semantic/instance segmentation, pose estimation, and tracking. Cattle, sheep/goats, pigs, and poultry were the major farm animal species of concern. In this research, preparations for system development, including camera settings, inclusion of variations for data recordings, choices of graphics processing units, image preprocessing, and data labeling were summarized. CNN architectures were reviewed based on the computer vision tasks in animal farming. Strategies of algorithm development included distribution of development data, data augmentation, hyperparameter tuning, and selection of evaluation metrics. Judgment of model performance and performance based on architectures were discussed. Besides practices in optimizing CNN-based computer vision systems, system applications were also organized based on year, country, animal species, and purposes. Finally, recommendations on future research were provided to develop and improve CNN-based computer vision systems for improved welfare, environment, engineering, genetics, and management of farm animals.

## 1. Introduction

Food security is one of the biggest challenges in the world. The world population is projected to be 9.2 billion in 2050 [1]. As livestock and poultry contribute to a large proportion (~30% globally) of daily protein intake through products like meat, milk, egg, and offal [2], animal production is predicted to increase accordingly to feed the growing human population. Based on the prediction by Yitbarek [3], 2.6 billion cattle, 2.9 billion sheep and goats, 1.1 billion pigs, and 37.0 billion poultry will be produced in 2050. As production is intensified to meet the increased demands, producers are confronted with increasing pressure to provide quality care for increasing number of animals per management unit [4]. This will become even more challenging with predicted labor shortages for farm jobs in the future [5]. Without quality care, some early signs of animal abnormalities may not be detected timely. Consequently, productivity and health of animals and economic benefits of farmers may be compromised [6]. Supportive technologies for animal farming are therefore needed for data collection and analysis and decision-making. Precision livestock farming (PLF) is the development and utilization of technologies to assist animal production and address challenges of farm stewardship.

The term “PLF” was proposed in the 1st European Conference on Precision Livestock Farming [7]. Since then, the concept has widely spread in animal production, and PLF-related technologies have been increasingly developed. Tools adopting the PLF concept feature continuous and real-time monitoring and/or big data collection and analysis that serve to assist producers in management decisions and provide early detection and prevention of disease and production inefficiencies [8]. PLF tools also offer objective measures of animal behaviors and phenotypes as opposed to subjective measures done by human observers [9,10], thus avoiding human observation bias. These tools can help to provide evidence-based strategies to improve facility design and farm management [11]. As these precision tools offer many benefits to advance animal production, efforts have been dedicated to developing different types of PLF tools. Among them, computer vision systems are ones of the popular tools utilized in animal sector. 

**Figure 1:**
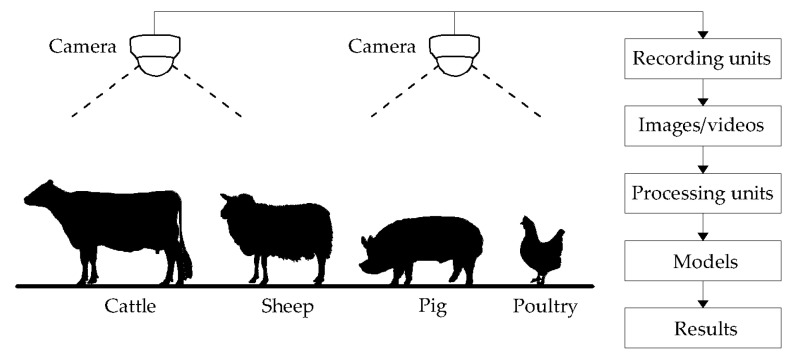
Schematic drawing of a computer vision system for monitoring animals.

The advantages of computer vision systems lie in non-invasive and low-cost animal monitoring. As such, these systems allow for information extraction with minimal external interferences (e.g., human adjustment of sensors) at an affordable cost [12]. The main components of a computer vision system include cameras, recording units, processing units, and models (Figure 1). In an application of a computer vision system, animals (e.g., cattle, sheep, pig, poultry, etc.) are monitored by cameras installed at fixed locations, such as ceilings and passageways, or onto mobile devices like rail systems, ground robots, and drones. Recording units (e.g., network video recorder or digital video recorder) acquire images or videos at different views (top, side, or front view) and various types (e.g., RGB, depth, thermal, etc.). Recordings are saved and transferred to processing units for further analysis. Processing units are computers or cloud computing servers [e.g., Amazon Web Servers, Azure of Microsoft, etc.]. In some real-time applications, recording and processing units may be integrated as the same units. Models in processing units are used to extract information of interest and determine the quality of results, thus being the core components in computer vision systems. 

Conventionally, processing models are created using image processing methods, machine learning algorithms, or combinations of the two. Image processing methods extract features from images via image enhancement, color space operation, texture/edge detection, morphological operation, filtering, etc. Challenges in processing images and videos collected from animal environments are associated with inconsistent illuminations and backgrounds, variable animal shapes and sizes, similar appearances among animals, animal occlusion and overlapping, and low resolutions [13]. These challenges considerably affect the performance of image processing methods and result in poor algorithm generalization for measuring animals from different environments. Machine learning-based techniques generally consist of feature extractors that transform raw data (i.e., pixel values of an image) into feature vectors and learning subsystems that regress or classify patterns in the extracted features. These techniques generally improve the generalizability but still cannot handle complex animal housing environments. In addition, constructing feature extractors requires laborious adjustment and considerable technical expertise [14], which limits wide applications of machine learning-based computer vision systems in animal farming.

Deep learning techniques, further developed from conventional machine learning, are representation-learning methods and can discover features (or data representation) automatically from raw data without extensive engineering knowledge on feature extraction [14]. This advantage makes the methods generalized in processing images from different animal farming environments. A commonly-used deep learning technique is the convolutional neural network (CNN). CNNs are artificial neural networks that involve a series of mathematical operations (e.g., convolution, initialization, pooling, activation, etc.) and connection schemes (e.g., full connection, shortcut connection, plain stacking, inception connection, etc.). The most significant factors that contribute to the huge boost of deep learning techniques are the appearance of large, high-quality, publicly-available datasets, along with the empowerment of graphics processing units (GPU), which enable massive parallel computation and accelerate training and testing of deep learning models [15]. Additionally, the development of transfer learning (a technique that transfers pretrained weights from large public datasets into customized applications) and appearance of powerful frameworks (e.g., TensorFlow, PyTorch, Theano, etc.) break down barriers between computer science and other sciences, including agriculture science, therefore CNN techniques are increasingly adopted for PLF applications. However, the status quo of CNN applications for animal sector has not been thoroughly reviewed, and questions remain to be addressed regarding the selection, preparation, and optimization of CNN-based computer vision systems. It is thus necessary to conduct a systematic investigation on CNN-based computer vision systems for animal farming to better assist precision management and future animal production.

Previous investigators have reviewed CNNs used for semantic segmentation [16], image classification [17], and object detection [18] and provided information on development of CNN-based computer vision systems for generic purposes, but none of these reviews has focused on agriculture applications. Some papers focused on CNN-based agriculture applications [19,20,21], but they were only related to plant production instead of animal farming. Okinda et al. [13] discussed parts of CNN-based applications in poultry farming, however, other species of farm animals were left out. Therefore, the objective of this study was to investigate and review CNN-based computer vision systems in animal farming. Specifically, Section 2 provides some general background knowledge (e.g., definitions of farm animals, computer vision tasks, etc.) to assist in the understanding of this research review. Section 3 summarizes preparations for collecting and providing high-quality data for model development. Section 4 organizes CNN architectures based on various computer vision tasks. Section 5 reviews strategies of algorithm development in animal farming. Section 6 studies performance judgment and performance based on architectures. Section 7 summarizes the applications by years, countries, animal species, and purposes. Section 8 briefly discusses and foresees trends of CNN-based computer vision systems in animal farming. Section 9 concludes the major findings of this research review. 

## 2. Study Background

In this section, we provide some background knowledge that is missed in Section 1 but deemed critical for understanding this research.

### 2.1. Definition of Farm Animals

Although “poultry” is sometimes defined as a subgroup under “livestock”, it is treated separately from “livestock” in this study according to the definition by the Food and Agriculture Organization of the United States [22]. Livestock are domesticated mammal animals (e.g., cattle, pig, sheep, etc.), and poultry are domesticated oviparous animals (e.g., broiler, laying hen, turkey, etc.). The investigated farm animals are as follows since they are major contributors for human daily food products.

- Cattle: a common type of large, domesticated, ruminant animals. In this case, they include dairy cows farmed for milk and beef cattle farmed for meat.
- Pig: a common type of large, domesticated, even-toed animals. In this case, they are sow farmed for reproducing piglets, piglet (a baby or young pig before it is weaned), and swine (alternatively termed pig) farmed for meat.
- Ovine: a common type of large, domesticated, ruminant animals. In this case, they are sheep (grazer) farmed for meat, fiber (wool), and sometimes milk, lamb (a young sheep), and goat (browser) farmed for meat, milk, and sometimes fiber (wool).
- Poultry: a common type of small, domesticated, oviparous animals. In this case, they are broiler farmed for meat, laying hen farmed for eggs, breeder farmed for reproducing fertilized eggs, and pullet (a young hen).

This review mainly focuses on monitoring the whole or parts of live animals in farms, since we intended to provide insights into animal farming process control. Other studies related to animal products (e.g., floor egg) or animal carcasses were not considered in this study.

### 2.2. History of Artificial Neural Networks for Convolutional Neural Networks in Evaluating Images/Videos

**Figure 2:**
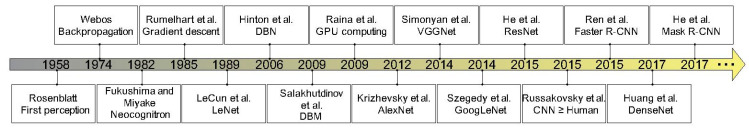
Some milestone events of development of artificial neural networks for convolutional neural networks in computer vision. DBN is deep brief network, GPU is graphics processing unit, DBM is Deep Boltzmann Machine, CNN is convolutional neural network, LeNet is a CNN architecture proposed by Yann LeCun, AlexNet is a CNN architecture designed by Alex Krizhevsky, VGGNet is Visual Geometry Group CNN, GoogLeNet is improved LeNet from Google, ResNet is residual network, faster R-CNN is faster region-based CNN, DenseNet is densely CNN, and mask R-CNN is mask region-based CNN. The CNN architectures after 2012 are not limited in this figure, and the selected ones are deemed influential and have at least 8000 citations in Google Scholar.

Development of artificial neural networks for CNNs or deep learning has a long history (Figure 2). In 1958, Rosenblatt [23] created the first perceptron that contained one hidden layer, activation function, and binary outputs to mimic how a brain perceived information outside. However, due to primitive computing equipment and an inability to accommodate solutions to non-linear problems, the development of machine learning did not appreciably advance. In 1974, Webos introduced backpropagation into a multi-layer perceptron in his PhD dissertation to address non-linearities [24], which made significant progress in neural network development. Rumelhart et al. [25] continued to research backpropagation theory and proposed critical concepts, such as gradient descent and representation learning. Fukushima and Miyake [26] further generated the Neocognitron Network that was regarded as the ancestor of CNNs. 

LeCun et al. [27] were the first to design a modern CNN architecture (LeNet) to identify handwritten digits in zip codes, and the network was reading 10–20% of all the checks in the US. That started the era of CNNs in computer visions. However, very deep neural networks suffered from the problems of gradients vanishing or exploding and could only be applied in some simple and organized conditions. Meanwhile, machine learning classifiers (e.g., support vector machine) outperformed CNN with regard to computational efficiency and ability to deal with complex problems [28]. To overcome the drawback of CNNs, Geoffrey E. Hinton, known as one of the godfathers of deep learning, insisted on the research and proposed an efficient learning algorithm (deep brief network) to train deep networks layer by layer [29]. The same research team also constructed the Deep Boltzmann Machine [30]. These ushered the “age of deep learning”. Additionally, Raina et al. [31] pioneered the use of graphics processing units (GPU) to increase the training speed of networks, which was 72.6 times faster at maximum than central processing unit (CPU) only. In the same year, the largest, publicly-available, annotated dataset, ImageNet, was created to encourage CNN development in computer vision [32]. With these well-developed conditions, Krizhevsky et al. [33] trained a deep CNN (AlexNet) to classify the 1.2 million high-resolution images in the 2012 ImageNet Classification Challenge and significantly improved the top-5 classification accuracy from 74.2% to 84.7%. 

Since then, artificial intelligence has entered its golden age. Excellent architectures for image classification from 2014 to 2017 were developed, such as Visual Geometry Group CNN (VGGNet) [34], improved LeNet from Google (Google, LLC, Mountain View, CA, USA) abbreviated as GoogLeNet [35], Residual Network (ResNet) and its variants [36], faster region-based CNN (faster R-CNN) [37], mask region-based CNN (mask R-CNN) [38], and Densely CNN (DenseNet) [39]. These CNNs dramatically improved algorithm performance in computer vision because of efficient mathematical operations (e.g., 3 × 3 kernel) and refined connection schemes (e.g., Inception modules, Shortcut connection, and Dense block, etc.). With these improvements, CNN can even outperform humans to classify millions of images [40]. 

### 2.3. Computer Vision Tasks

**Figure 3:**
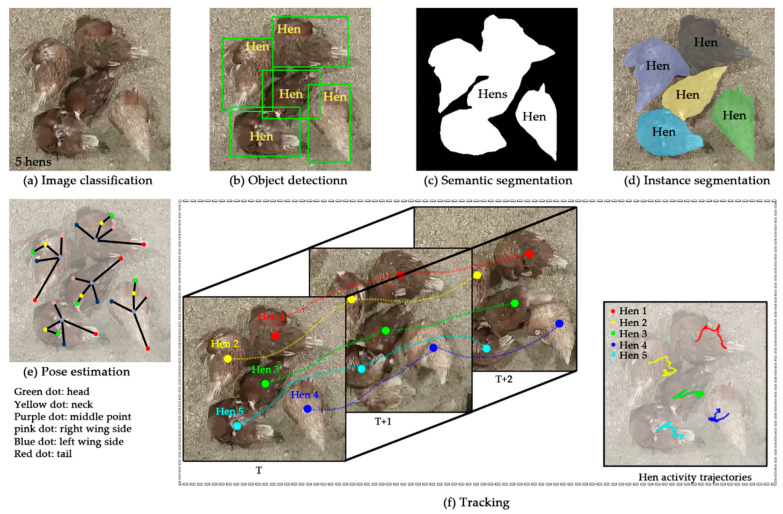
Example illustrations of six computer vision tasks. The semantic and instance segmentations were combined as semantic/instance segmentation due to many similarities, resulting in the major five computer vision tasks throughout the study.

Current public datasets are mainly for six computer vision tasks or challenges: image classification, object detection, semantic segmentation, instance segmentation, pose estimation, and tracking (Figure 3), so that researchers can develop corresponding models to conduct the tasks and tackle the challenges. As semantic segmentation and instance segmentation have many similarities, they were combined as semantic/instance segmentation, resulting in the major five computer vision tasks in this study. Image classification is to classify an image as a whole, such as five hens in an image (Figure 3a) [17]. Object detection is to detect and locate individual objects of concern and enclose them with bounding boxes (Figure 3b) [18]. Semantic segmentation is to segment objects of concern from a background (Figure 3c) [16]. Instance segmentation is to detect and segment object instances marked as different colors (Figure 3d) [16]. As semantic/instance segmentation assigns a label to each pixel of an image, it is also categorized as a dense prediction task. Pose estimation is to abstract individual objects into key points and skeletons and estimate poses based on them (Figure 3e) [15]. Tracking is to track objects in continuous frames based on changes of geometric features of these objects, and typically object activity trajectories are extracted (Figure 3f) [15]. In this research summary, even though some applications may involve multiple computer vision tasks during intermediate processes, they were categorized only based on ultimate outputs. 

### 2.4. “Convolutional Neural Network-Based” Architecture

**Figure 4:**
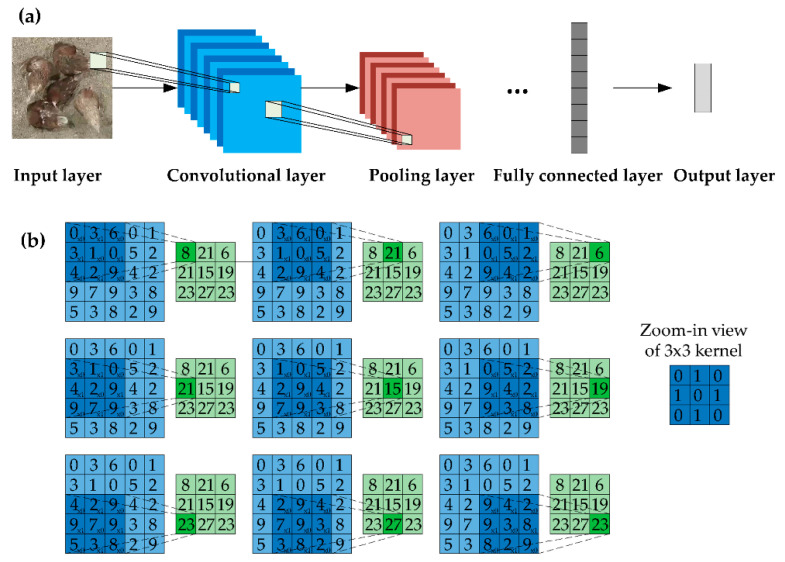
Example illustrations of (**a**) a convolutional neural network and (**b**) a convolution with kernel size of 3 × 3, stride of 1, and no padding.

The “CNN-based” means the architecture contains at least one layer of convolution for processing images (Figure 4a,b). In fact, the investigated applications may include other advanced deep learning techniques in computer vision, such as long short-term memory (LSTM) and generative adversarial network (GAN).

### 2.5. Literature Search Term and Search Strategy

**Figure 5:**
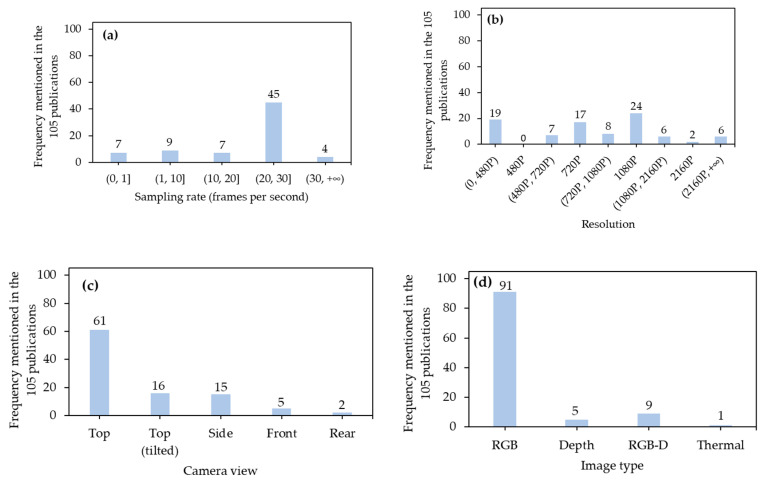

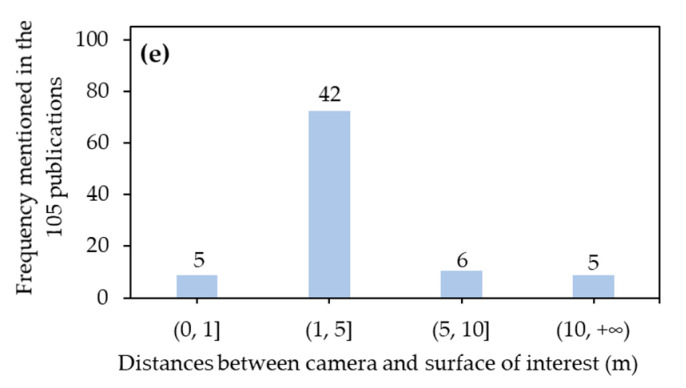
Frequency mentioned in the 105 publications corresponding to different camera settings: (**a**) sampling rate; (**b**) resolution; (**c**) camera view; (**d**) image type; and (**e**) distances between camera and surface of interest. (**a**,**b**,**d**): number near a round bracket is not included in specific ranges while number near square bracket is. (**b**): 480P is 720 × 480 pixels, 720P is 1280 × 720 pixels, 1080P is 1920 × 1080 pixels, and 2160P is 3840 × 2160 pixels. (**d**): RGB is red, green, and blue; and RGB-D is RGB and Depth.

**Figure 6:**
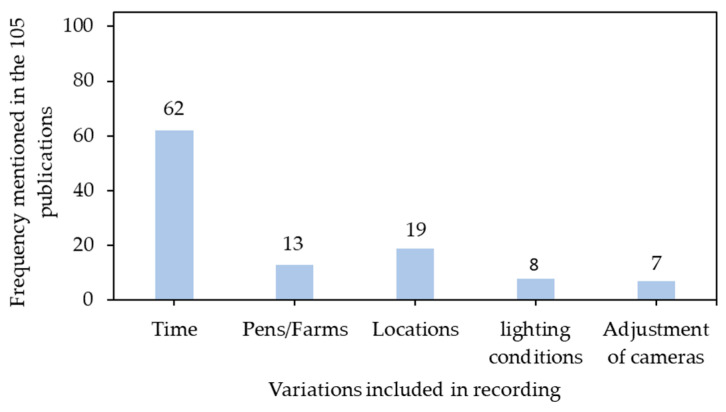
Frequency mentioned in the 105 publications corresponding to different variations included in data recording.

**Figure 7:**
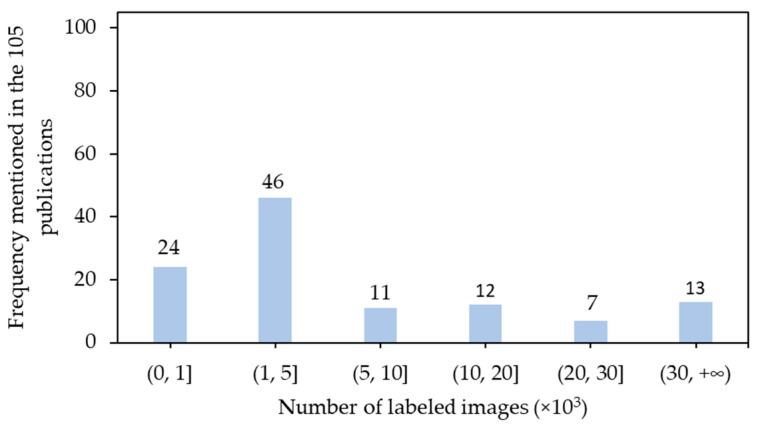
Frequency mentioned in the 105 publications corresponding to number of labeled images.

**Figure 8:**
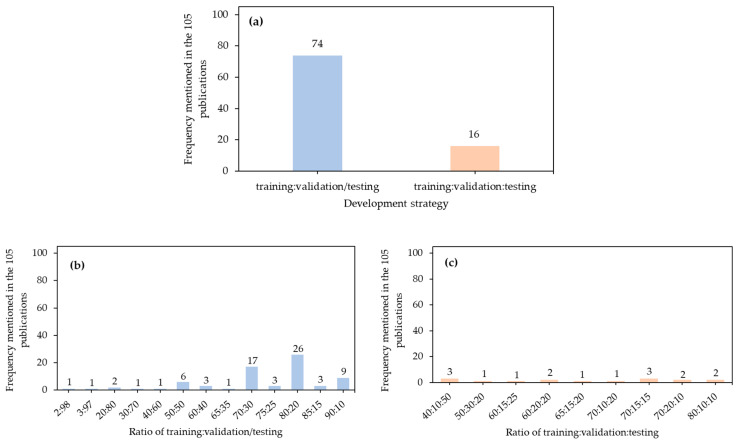
Frequency of development strategies in the 105 publications. (**a**) Frequency of the two development strategy; (**b**) frequency of different ratios of training:validation/testing; and (**c**) frequency of different ratios of training:validation:testing.

**Figure 9:**
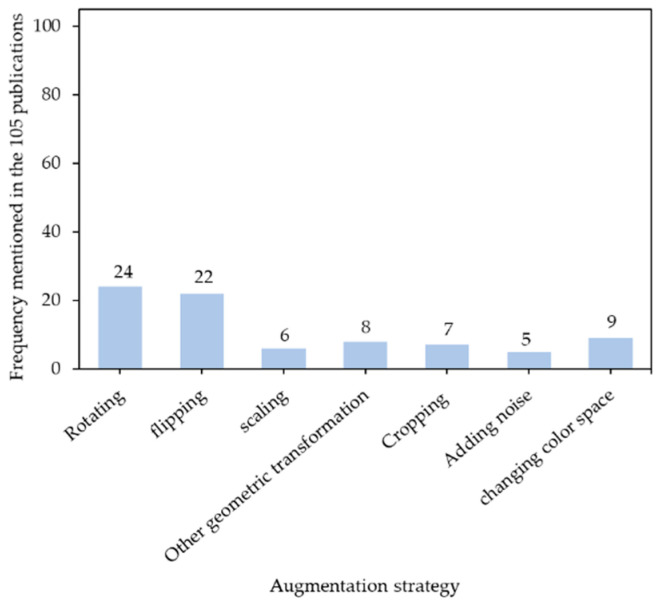
Frequency of different augmentation strategies in the 105 publications. Other geometric transformation includes distorting, translating, shifting, reflecting, shearing, etc.

**Figure 10:**
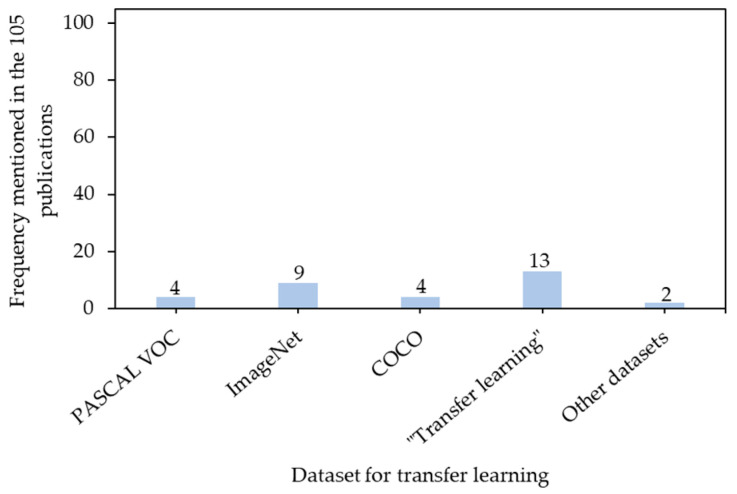
Frequency of different datasets mentioned in the 105 publications. PASCAL VOC is PASCAL visual object class. COCO is common objects in context. “Transfer learning” means the publications only mentioned “transfer learning” rather than specified datasets for transfer learning. Other datasets are motion analysis and re-identification set (MARS) and action recognition dataset (UCF101).

**Figure 11:**
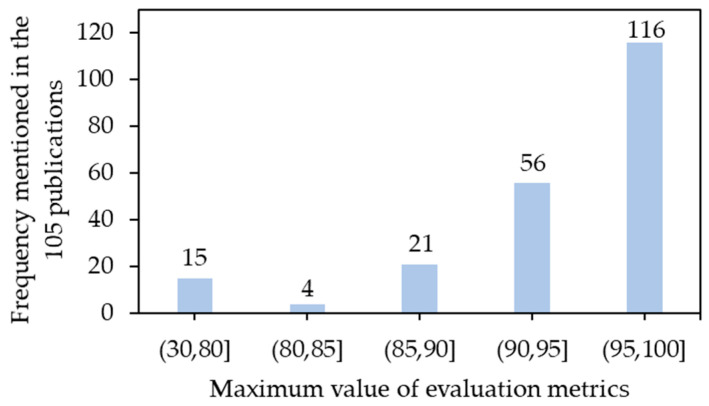
Frequency of value of evaluation metrics in the 105 publications. Metrics include, but are not limited to, accuracy, specificity, recall, precision, average precision, mean average precision, F1 score, and intersection over union. Number near a round bracket is not included in specific ranges while number near square bracket is.

**Figure 12:**
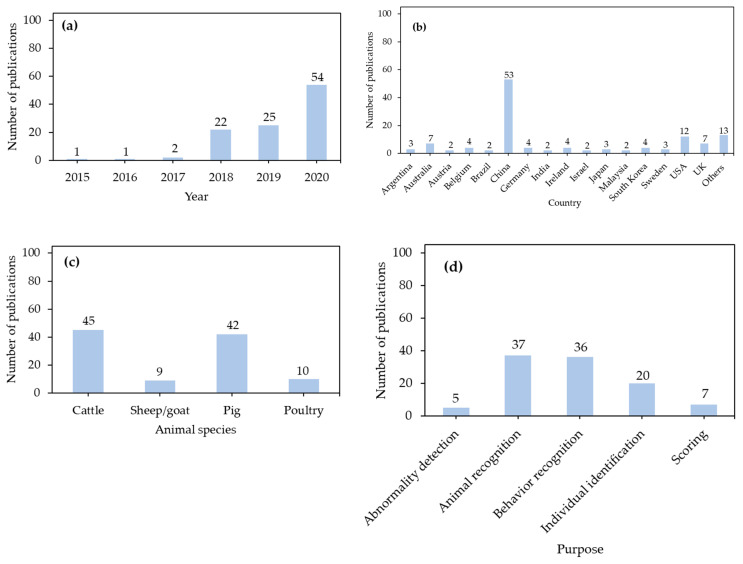
Number of publications based on (**a**) year, (**b**) country, (**c**) animal species, and (**d**) purpose. One publication can include multiple countries and animal species. “Others” are Algeria, Philippines, Nigeria, Italy, France, Turkey, New Zealand, Indonesia, Egypt, Spain, Norway, Saudi Arabia, and Switzerland.

For this investigation, the literature was collected from six resources: Elsevier ScienceDirect, IEEE Xplore Digital Library, Springer Link, ASABE Technical Library, MDPI Database, and Google Scholar. The keywords for the retrieval included “deep learning”, “CNN”, “convolutional neural network”, “computer vision”, “livestock”, “poultry”, “cow”, “cattle”, “sheep”, “goat”, “pig”, “laying hen”, “broiler”, and “turkey”. By 28 October, 2020, a total of 105 references were found and summarized in this study. It should be noted that there can be missing information or multiple settings in specific references, therefore, the sum of number of reviewed items in Figure 5, Figure 6, Figure 7, Figure 8, Figure 9, Figure 10, Figure 11 and Figure 12 could not exactly be 105. 

## 3. Preparations

The preparations contain camera setups, inclusion of variations in data recording, GPU selection, image preprocessing, and data labeling, since these were deemed to significantly affect data quality and efficiency of system development. 

### 3.1. Camera Setups

In this section, sampling rate, resolution, camera view, image type, and distance between camera and surface of interest with regard to setting up cameras are discussed.

#### 3.1.1. Sampling Rate

A sampling rate determines the number of frames collected in one second (fps) (Figure 5a). High sampling rates serve to reduce blurred images of target animals and capture prompt movement among frames but may lead to recording unnecessary files and wasting storage space [41,42]. The most commonly-used sampling rates were 20–30 fps, since these can accommodate movement capture of farm animals in most situations [41]. The lowest sampling rate was 0.03 fps [43]. In that study, a portable camera was used, and images were saved into a 32-GB SD card. The low sampling rate can help to reduce memory usage and extend recording duration. The highest sampling rate reached 50 fps [44], since the authors intended to continuously record the movement of the entire body of each cow to make precise predictions of lameness status. 

#### 3.1.2. Resolution

Resolution refers to the number of pixels in an image (Figure 5b). High resolutions can ensure an elevated level of details in images of target animals but it can increase the file size and storage space needed [45]. The 1080P (1920 × 1080 pixels) and 720P (1280 × 720 pixels) resolutions were common in the literature, likely due to the preset parameters and capabilities found in current video recorders. It should be noted that even though higher resolution images are more detailed, higher resolutions did not necessarily result in better detection performance. Shao et al. [45] tested the image resolutions of 416 × 416, 736 × 736, 768 × 768, 800 × 800, 832 × 832, and 1184 × 1184 pixels. The results indicated that the resolution of 768 × 768 pixels achieved the highest detection performance. 

#### 3.1.3. Camera View 

A camera view is determined by parts of interest of target animals (Figure 5c). Although capturing the top parts of animals with a camera right above them (top view) can capture many pose details of animals and be favorable for behavior recognition thus being widely adopted [46], other camera views were also necessary for specific applications. Tilting cameras on top can provide a perspective view and include animals on far sides [47]. As for some large farm animals (e.g., cattle), their ventral parts typically had larger surface area and more diverse coating patterns than their dorsal parts. Therefore, a side view was favorable for individual cattle recognition based on their coating pattern [48]. A front view was required for face recognition of farm animals [49], and rear-view images were used to evaluate the body conditions of farm animals based on their features of rear parts [50].

#### 3.1.4. Image Type 

Types of images used in the 105 publications included RGB (red, green, and blue), depth, RGB + depth, and thermal (Figure 5d). The RGB images were the most prevalent. Most of the applications transferred CNN architectures pretrained from other publicly-available datasets. RGB images were built and annotated in these datasets, and using RGB images may improve the efficiency of transfer learning [51]. Including depth information may further improve the detection performance. Zhu et al. [52] paired RGB and depth images and input them simultaneously into a detector. The combination of these two types of images outperformed the use of RGB imaging or depth imaging singly. Infrared thermal imaging took advantage of temperature differences between background and target objects, and it achieved high detection performance for some animal parts (e.g., eyes and udders of cows) with relatively high temperature [53]. However, in many cases, thermal images are not suitable for analysis given the variability in emissivity due to environmental conditions and user errors in adjusting for varying emissivity on targets.

#### 3.1.5. Distance between Camera and Surface of Interest 

Distances between a camera and surface of interest determine how many pixels represent target objects (Figure 5e). Generally, a closer distance resulted in more pixels and details representing farm animals, although cameras with closer distances to target animals may be more likely to be damaged or contaminated by them [54]. The distances of 1-5 m were mostly utilized, which were fitted to dimensions of modern animal houses and simultaneously advantageous on capturing most details of target animals. For those applications requiring clear and obvious features of target objects (e.g., faces of animals), close distances (e.g., 0.5 m) were preferred [55]. An unmanned aerial vehicle (UAV) was utilized to count animals at long range distances (e.g., 80 m away from animals), which minimized the interference of detection [56]. It should be highlighted that UAVs generate wind and sound at short ranges, which may lead to stress for animals [57]. As such, gradual changes in distances at which images are captured should be considered. 

### 3.2. Inclusion of Variations in Data Recording

Current CNN architectures have massive structures relative to connection schemes, parameters, etc. Sufficient variations in image contents should be fed into CNN architectures to increase the robustness and performance in unseen situations. A common strategy of including variations involved a continuous temporal recording of images (e.g., sections of a day, days, months, and seasons) (Figure 6). Farm animals are dynamic and time-varying [58], and sizes, number of animals, and individuals can be different in a recording area across a timeline, which creates variations for model development. Additionally, temporal acquisition of outdoor images also introduced variations of background [54]. Animal confinement facilities and facility arrangements vary across farms and production systems [59,60], therefore, moving cameras or placing multiple cameras in different locations within the same farm/facility can also capture various backgrounds contributing to data diversity [61,62]. Recording images under different lighting conditions (e.g., shadow, sunlight, low/medium/high light intensities, etc.) can also supply diverse data [54]. Additionally, adjusting cameras is another strategy of including variations and may change shapes, sizes, and poses of target animals in recorded images, such as moving cameras around target animals [63], adjusting distances between cameras and target animals [55], modifying heights and tilting angles of cameras [64], etc.

### 3.3. Selection of Graphics Processing Units

Graphics processing units assist in massive parallel computation for CNN model training and testing and are deemed as core hardware for developing models. Other influential factors, such as processor, random-access memory (RAM), and operation systems, were not considered in this case since they did not affect computation of CNN as significantly as GPU did. To provide brief comparisons among different GPU cards, we also retrieved their specifications from versus (https://versus.com/en, accessed on 31 October 2020), Nvidia (https://www.nvidia.com/en-us/, accessed on 31 October 2020), and Amazon (https://www.amazon.com/, accessed on 31 October 2020) and compared them in Table 1. Computer unified device architecture (CUDA) cores are responsible for processing all data that are fed into CNN architectures, and higher number of CUDA cores is more favorable for parallel computing. Floating point performance (FPP) reflects speed of processing floating points, and a larger FPP means a faster processing speed. Maximum memory bandwidth (MMB) is the theoretical maximum amount of data that the bus can handle at any given time and determines how quickly a GPU can access and utilize its framebuffer. 

Among all GPU products in Table 1, the NVIDIA GeForce GTX 1080 TI GPU was mostly used in developing computer vision systems for animal farming due to its affordable price and decent specifications. Although the NVIDIA company developed GeForce RTX 30 series for faster deep learning training and inference, due to their high price, the GeForce GTX 10 series and GeForce RTX 20 series may still be popular, balancing specifications and price. NVIDIA Tesla series (except for NVIDIA Tesla P100) were inexpensive and utilized in previous research [43], but they retired after May, 2020. NVIDIA Quadro series were also used previously but may not be recommended, because their price was close to that of NVIDIA GeForce GTX 1080 TI while number of CUDA cores, FPP, and MMB were lower than the latter. NVIDIA Jetson series may not outperform other GPUs with regard to the specifications listed in Table 1, but they were cheap, lightweight, and suitable to be installed onto UAVs or robots [89], which can help to inspect animals dynamically. Some research trained and tested some lightweight CNN architectures using CPU only [64,105,106,107], which is not recommended because training process was extremely slow and researchers may spend long time receiving feedback and making decision modifications [108]. If local machines are not available, researchers can rent cloud servers (e.g., Google Colab) for model development [54,104]. However, privacy concerns may limit their use in analysis of confidential data.

### 3.4. Image Preprocessing

Processing images can improve image quality and suitability before model development [89]. In this section, the preprocessing solutions include selection of key frames, balance of datasets, adjustment of image channels, image cropping, image enhancement, image restoration, and image segmentation [109]. Although some research also defined image resizing [76] and data augmentation [110] as image preprocessing, they were included either in CNN architectures or in model training processes, thus not being discussed in this section.

#### 3.4.1. Selection of Key Frames

As mentioned in Section 3.2, most applications adopted continuous monitoring. Although such a strategy can record as many variations as possible, it may result in excessive files for model development and decrease development efficiency. General solutions are to manually remove invalid files and retain informative data for the development. Examples of invalid files include blurred images, images without targets or only with parts of targets, and images without diverse changes [11,51,80,90,107]. Some image processing algorithms were available to compare the differences between adjacent frames or between background frames and frames to be tested and capable of automatically ruling out unnecessary files. These included, but were not limited to, Adaptive Gaussian Mixture Model [111], Structural Similarity Index Model [49,70], Image Subtraction [54], K-Mean Clustering [97], Absolute Histogram Difference-Based Approach [112], and Dynamic Delaunay Graph Clustering Algorithm [65].

#### 3.4.2. Class Balance in Dataset 

Imbalanced datasets contain disproportionate amount of data among classes and may result in biased models which infer classes with small-proportion training data less accurately [107,113]. The class imbalance can be produced during recording of different occurrence frequencies of target objects (e.g., animal behaviors, animals) [111,113]. A common way to correct the imbalance is to manually and randomly balance the amount of data in classes [47,54,114]. Alvarez et al. [108] complemented confidence of inference of minority class via increasing class weights for them. Zhu et al. [52] set a larger frame interval for sampling images containing high-frequency behaviors. Another alternative method was to synthesize and augment images for minority classes during training [57]. Details of data augmentation are discussed later in Section 5.2.

#### 3.4.3. Adjustment of Image Channels

An RGB image contains three channels and reflects abundant features in color spaces. However, lighting conditions in farms are complex and diverse, and color-space patterns learned from development datasets may not be matched to those in real applications, which may lead to poor generalization performance [115]. One solution was to convert RGB images (three channels) into grayscale images (one channel) [48,55,70,88,116], so that attention of models can be diverted from object colors to learning patterns of objects. Additionally, hue, saturation, and value (HSV) imaging was not as sensitive to illumination changes as RGB imaging and may be advantageous on detecting characteristics of colorful target objects [114].

#### 3.4.4. Image Cropping 

In an image, representations of background are sometimes much more than those of target objects, and models may learn many features of no interest and ignore primary interest [57]. One strategy to improve efficiency is to crop an image into regions of interest before processing. The cropping can be based on a whole body of an animal [80,117,118], parts (e.g., face, trunk) of an animal [55,107], or areas around facilities (e.g., enrichment, feeder, and drinker) [86,114,119]. As for some large images, cropping them into small and regular pieces of images can reduce computational resources and improve processing speed [56,64,106,120].

#### 3.4.5. Image Enhancement 

Image enhancement highlights spatial or frequency features of images as a whole, so that the models can concentrate on these patterns. Direct methods for image enhancement in frequency domain are to map predefined filters onto images with frequency transformations and retain features of interest. These include, but are not limited to, high/low pass filters to sharpen/blurred areas (e.g., edges) with sharp intensity changes in an image [108] and bilateral filters to preserve sharp edges [53]. As for enhancement in spatial domain, manipulations in histograms are commonly used to improve brightness and contrast of an image. Examples include histogram equalization [73,121], adaptive histogram equalization [52,67], and contrast limited adaptive histogram equalization [70,89,122]. Other intensity transformation methods (e.g., adaptive segmented stretching, adaptive nonlinear S-curve transform, and weighted fusion) were also used to enhance the intensities or intensity changes in images [53].

#### 3.4.6. Image Restoration 

Target objects in an image can be disproportional regarding sizes and/or shapes because of projective distortion caused by various projection distances between objects and cameras with a flat lens [123]. To correct the distortion and uniform objects in an image, some standard references (e.g., straight line, marker, and checkerboard) can be annotated in images [79,107,124,125], and then different transformation models (e.g., isometry, similarity, affine, and projective) can be applied to make the whole images match the respective references [123]. In addition, some restoration models can be applied to remove the noises processed during data acquisition, encoding, and transmission and to improve image quality [126]. Median filtering that averages intensities based on neighboring pixels was used in general [52,110,122], and other models that included two-dimensional gamma function-based image adaptive correction algorithm and several layers of neural networks were also available for the denoising [63,126,127]. Some images contain natural noises that are hard to be denoised. Adding known noises (with predefined mean and variance) including Gaussian, salt, and pepper noises may improve the denoising efficiency [63,126]. As for depth sensing, uneven lighting conditions and object surfaces, dirt, or dust may result in holes within an object in an image [115,128]. The restoring strategies were interpolation (e.g., spatiotemporal interpolation algorithms) [73,128], interception (e.g., image center interception) [110], filtering (e.g., median filtering) [110,122], and morphological operation (e.g., closing) [122].

#### 3.4.7. Image Segmentation 

In some applications (e.g., cow body condition scoring based on edge shapes of cow body [101]), background may contain unnecessary features for inference and decrease detection efficiencies. Image segmentation is conducted to filter out background and preserve features of interest. Some common segmentation models are Canny algorithm [101,108], Otsu thresholding [73], and phase congruency algorithm [115].

### 3.5. Data Labeling

Current CNNs in computer vision are mostly supervised learning techniques. Labels that are input into model training influence what patterns models learn from an image. For some applications of animal species recognition, professional labeling knowledge in animals may not be required since labelers only needed to distinguish animals from their respective backgrounds [59]. However, as for behavior recognition, animal scientists were generally required to assist in behavior labeling, because labels of animal behaviors should be judged accurately by professional knowledge [102]. A sole well-trained labeler was typically responsible for completion of all labeling, because labels may be influenced by judgment bias from different individuals [129]. However, multiple labelers involved can serve to expedite the labeling process. Under that circumstance, a standard protocol containing clear definitions of labels should be built so that labelers can follow a uniform approach. Mutual reviews among labelers may also be required to minimize the subjective errors [51]. Additionally, to supply models with completed and accurate features of target objects, animals at edges of images were labeled if over 50% of their bodies were visible [120], or images were removed from development datasets if only small parts of animal bodies were presented [107].

Appropriate labeling tools can increase conveniences in producing labels and be specific for different computer vision tasks. Available labeling tools for different computer vision tasks are presented in Table 2. Labels of image classification are classes linked with images, and the labeling is done via human observation. No labeling tool for image classification was found in the 105 references. As for object detection, LabelImg is the most widely-used image labeling tool and can provide labels in Pascal Visual Object Classes format. Other tools (such as Image Labeler and Video Annotator Tool from Irvine, California) for object detection can also provide label information of bounding boxes of target objects along with class names [130,131]. Tools for semantic/instance segmentation can assign attributes to each pixel. These included Graphic [92], Supervisely [104], LabelMe [64,90,112], and VGG Image Annotator [46,51]. Labels of pose estimation generally consist of a series of key points, and available tools for pose estimation were DeepPoseKit [97] and DeepLabCut [132]. Tracking labels involve assigned IDs or classes for target objects through continuous frames, and the tools for tracking were Kanade-Lucas-Tomasi tracker [88], Interact Software [72], and Video Labeler [68]. It should be noted that references of the aforementioned tools only indicate sources of label tool applications in animal farming rather than developer sources, which can be found in Table 2.

Another key factor to be considered is the number of labeled images. Inadequate number of labeled images may not be able to feed models with sufficient variations and result in poor generalization performance, while excessively labeled images may take more time to label and obtain feedbacks during training. A rough rule of thumb is that a supervised deep learning algorithm generally achieves good performance with around 5000 labeled instances per category [147], while 1000–5000 labeled images were generally considered in the 105 references (Figure 7). The least number was 33 [50], and the largest number was 2270250 [84]. Large numbers of labeled images were used for tracking, in which continuous frames were involved. If there were high number of instances existing in one image, a smaller amount of images was also acceptable on the grounds that sufficient variations may occur in these images and be suitable for model development [96]. Another strategy to expand smaller number of labeled images was to augment images during training [71]. Details of data augmentation are introduced in Section 5.2. 

## 4. Convolutional Neural Network Architectures 

Convolutional neural networks include a family of representation-learning techniques. Bottom layers of the networks extract simple features (e.g., edges, corners, and lines), and top layers of the networks infer advanced concepts (e.g., cattle, pig, and chicken) based on extracted features. In this section, different CNN architectures used in animal farming are summarized and organized in terms of different computer vision tasks. It should be noted that CNN architectures are not limited as listed in the following section. There are other more advanced network architectures that have not been applied in animal farming.

### 4.1. Architectures for Image Classification 

Architectures for image classification are responsible for predicting classes of images and are the most abundant among the five computer vision tasks. Early researchers explored the CNN with shallow networks and feed-forward connections (e.g., LeNet [27] and AlexNet [33]). These networks may not work well in generalization for some complex problems. To improve the performance, several convolutional layers were stacked together to form very deep networks (e.g., VGGNet [34]), but it introduced the problems of high computation cost and feature vanishing in backpropagation of model training. Several novel ideas were proposed to improve the computational efficiency. For example, directly mapping bounding boxes onto each predefined grid and regressing these boxes to predict classes (You only look once, YOLO [148]); expanding networks in width rather than in depth (GoogLeNet [35]); and separating depth-wise convolutions to reduce depth of feature maps (MobileNet [149]). To reduce feature vanishing, different types of shortcut connections were created. For instance, directly delivering feature maps to next layers (ResNet50 [36]) or to every other layer in a feed-forward fashion (Densely connected network, DenseNet [39]). Building deep CNNs still required engineering knowledge, which may be addressed by stacking small convolutional cells trained in small datasets [150]. 

The ResNet50 [69,76,120,151] and VGG16 [49,76,101,107,120,151] were the most popular architectures used in animal farming (Table 3). The former may be an optimal model balancing detection accuracy and processing speed thus being widely used. The latter was one of the early accurate models and widely recognized in dealing with large image datasets. Sometimes, single networks may not be able to classify images from complex environments of animal farming, and combinations of multiple models are necessary. The combinations had three categories. The first one was to combine multiple CNNs, such as YOLO + AlexNet [151], YOLO V2 + ResNet50 [98], and YOLO V3 + (AlexNet, VGG16, VGG19, ResNet18, ResNet34, DenseNet121) [126]; the second one was to combine CNNs with regular machine learning models, such as fully connected network (FCN) + support vector machine (SVM) [68], Mask region-based CNN (mask R-CNN) + kernel extreme learning machine (KELM) [90], Tiny YOLO V2 + SVM [121], VGG16 + SVM [49], YOLO + AlexNet + SVM [74], and YOLO V3 + [SVM, K-nearest neighbor (KNN), decision tree classifier (DTC)] [152]; and the third one was to combine CNNs with other deep learning techniques, such as [Convolutional 3 dimension (C3D), VGG16, ResNet50, DenseNet169, EfficientNet] + long short-term memory (LSTM) [84], Inception V3 + bidirectional LSTM (BiLSTM) [41], and Inception V3 + LSTM [153], YOLO V3 + LSTM [152]. In all these combinations, CNN typically played roles of feature extractors in the first stage, and then other models utilized these features to make classifications. Because of excellent performance, the abovementioned CNNs were designed as feature extractors and embedded into architectures in the rest of computer vision tasks. 

### 4.2. Architectures for Object Detection 

Object detection architectures are categorized as fast detection networks, region-based networks, and shortcut connection networks (Table 4). The first category mainly consists of single shot detector (SSD [173]) family and YOLO [148] family. The SSD is a feed-forward network with a set of predefined bounding boxes at different ratios and aspects, multi-scale feature maps, and bounding box adjustment. It can detect objects at a 59-fps speed but may have low detection accuracy. Later the accuracy of the network was improved by adjusting receptive fields based on different blocks of kernels (RFBNetSSD [174]). YOLO separates images into a set of grids and associates class probabilities with spatially separated bounding boxes. It can achieve general detection speed of 45 fps and extreme speed of 155 fps but, like SSD, suffered from poor detection accuracy. The accuracy can be improved by adding multi-scale feature maps and lightweight backbone and replacing fully connected (FC) layers with convolutional layers (YOLO V2 [171]), using logistic regressors and classifiers to predict bounding boxes and introducing residual blocks (YOLO V3 [175]), or utilizing efficient connection schemes and optimization strategies (e.g., weighted residual connections, cross stage partial connections, cross mini-batch normalization, self-adversarial training, and mish activation for YOLO V4 [176]).

The second category is to initially propose a series of region proposals and then use classifiers to classify these proposals. The first version of region-based network (R-CNN [185]) proposed 2000 candidate region proposals directly from original images, which consumed much computational resource and time (~0.02 fps). Instead of directly producing proposals from images, fast R-CNN [195] first produced feature maps and then generated 2000 proposals from these maps, which can increase the processing speed to ~0.4 fps but was still not optimal. Faster R-CNN [37] used region proposal network (RPN) to generate proposals from feature maps and then used the proposals and same maps to make a prediction, with which processing speed rose to 5 fps. Then the region-based network improved its accuracy by pooling regions with region of interest (ROI) align instead of max/average pooling and adding another parallel network (FCN) at the end (Mask R-CNN [38]). Or its processing speed was improved by replacing FC layers with convolutional layers and using the position-sensitive score map (R-FCN [189]).

The third category is shortcut connection network, which can reduce feature vanishing during training. The first network using shortcut connection was ResNet [36], in which the shortcut connection was built between two adjacent blocks. Then the shortcut connection was extended to include multiple connections in depth (DenseNet [39]) or in width (ResNeXt [192]).

The abovementioned networks are efficient networks from different perspectives of views (e.g., processing speed or accuracy) and widely utilized in animal farming. Among them, SSD, YOLO V3, and faster R-CNN were mostly applied, since the former two had advantages in processing speed while the last one was advantageous on tradeoffs of processing speed and accuracy. In some relatively simple scenarios (e.g., optimal lighting, clear view), combinations of several shallow networks (i.e., VGG + CNN) may achieve good performance [124]. However, in some complex cases (e.g., real commercial environments), single networks may not work well, and combining multiple networks (i.e., UNet + Inception V4 [196], VGGNet + SSD [110]) can accordingly increase model capacity to hold sufficient environmental variations. Even with the same models, a parallel connection to form a two-streamed connection may also boost detection performance [52]. 

### 4.3. Architectures for Semantic/Instance Segmentation 

Modern CNN architectures for image segmentation mainly consist of semantic and instance segmentation networks (Table 5). Semantic models generally have encoders for downsampling images into high-level semantics and decoders for upsampling high-level semantics into interpretable images, in which only regions of interest are retained. The first semantic model was FCN [197]. Although it introduced the advanced concept of encoder–decoder for segmentation, it required huge amount of data for training and consumed many computational resources. It used repeated sets of simple convolution and max pooling during encoding and lost much spatial information in images, resulting in low resolution of images or fuzzy boundaries of segmented objects. To address the issues of exhausting training, UNet [198] was created based on contrasting and symmetric expanding paths and data augmentation, which required very few images to achieve acceptable segmentation performance. There were two types of networks to improve segmentation accuracy. One was fully convolutional instance-aware semantic segmentation network (FCIS [199]), and it combined position-sensitive inside/outside score map and jointly executed classification and segmentation for object instances. The other one was DeepLab [200]. It used Atrous convolution to enlarge a field of view of filters, Atrous spatial pyramid pooling to robustly segment objects at multiple scales, and a fully connected conditional random field algorithm to optimize boundaries of segmented objects. As for issues of intensive computational resources, the efficient residual factorized network (ERFNet [201]) optimized its connection schemes (residual connections) and mathematical operations (factorized convolutions). It retained remarkable accuracy (69.7% on Cityscapes dataset) while achieved high processing speed of 83 fps in a single Titan X and 7 fps in the Jetson TX1. Development of instance segmentation models is slower than semantic segmentation models, probably because they need more computational resources and, currently, may be suboptimal for real-time processing. Regardless of the processing speed, current instance segmentation models due to deeper and more complex architectures had more compelling segmentation accuracy than semantic segmentation models. One of the popular instance segmentation models was the mask R-CNN [38], which implemented object classification, object detection, and instance segmentation parallelly. Its segmentation accuracy can be further improved by adding another network (mask intersection over union network, “mask IOU network”) to evaluate extracted mask quality (Mask Scoring R-CNN [202]). 

The DeepLab [200], UNet [198], and Mask R-CNN [38] were applied in animal farming quite often because of their optimized architectures and performance as mentioned above. Combining segmentation models with simple image processing algorithms (e.g., FCN + Otsu thresholding [67]) can be helpful in dealing with complex environments in animal farming. Sometimes, such a combination can be simplified via using lightweight object detection models (e.g., YOLO) to detect objects of concern and simple image processing algorithms (e.g., thresholding) to segment objects enclosed in bounding boxes [82,208]. 

### 4.4. Architectures for Pose Estimation 

Few architectures for detecting poses of farm animals exist, and most of them are cascade/sequential models (Table 6). Pose estimation models are mainly used for humans and involve a series of key point estimations and key point associations. In this section, existent models of pose estimation of animal farming are organized into two categories. The first one was heatmap-free network. The representative one was DeepPose [210], which was also the first CNN-based human pose estimation model. It regressed key points directly from original images and then focused on regions around the key points to refine them. Although DeepLabCut [211] also produced key points from original images, it first cropped ROI for the key point estimation and adopted residual connection schemes, which speeded up the process and was helpful for training. Despite being optimized, directly regressing key points from original images was not an efficient way. A better method may be to generate a heatmap based on key points and then refine the locations of the key points. For the heatmap-based networks, convolutional part heatmap regression (CPHR [212]) can first detect relevant parts, form heatmap around them, and then regress key points from them; convolutional pose machines (CPMs [213]) had sequential networks and can address gradient vanishing during training; and Hourglass [214] consisted of multiple stacked hourglass modules which allow for repeated bottom-up, top-down inference. These are all optimized architectures for pose estimation. 

Among the existent models (Table 6), Hourglass [214] was the most popular in animal farming because of efficient connection schemes (repeated hourglass modules and ResNets) and remarkable breakthroughs of replacing regressing coordinates of key points with estimating heatmaps of these points. However, single networks may not be able to handle multiple animals in complex farm conditions, such as adhesive animals, occlusions, and various lighting conditions. To deal with those, some investigators firstly used CNN (e.g., FCN) to detect key points of different animals, then manipulated coordinates among these detected key points (e.g., creating offset vector, estimating maximum a posteriori), and finally associated key points from the same animals using extra algorithms (e.g., iterative greedy algorithm, distance association algorithm, etc.) [42,61,77].

### 4.5. Architectures for Tracking 

There are a few tracking models used in animal farming. Among the four existent tracking models listed in Table 7, the two-stream network [221] was the first tracking model proposed in 2014. It captured complementary information on appearance from still frames using several layers of convolutional networks and tracked the motion of objects between frames using optical flow convolutional networks. Then in 2015, long-term recurrent convolutional networks (LRCN) [222] were created. They generally consist of several CNNs (e.g., Inception modules, ResNet, VGG, Xception, etc.) to extract spatial features and LSTM to extract temporal features, which is called “doubly deep”. Then an extremely fast, lightweight network (Generic Object Tracking Using Regression Network, GOTURN) was built and can achieve 100 fps for object tracking. The network was firstly trained with datasets containing generic objects. Then during the testing, ROIs of current and previous frames were input together into the trained network, and location of targets was predicted continuously. Dating back to 2019, the SlowFast network tracked objects based on two streams of frames, in which one was in low frame rate, namely slow pathway and the other was in high frame rate, namely high pathway. These are useful architectures to achieve performance of interest for target tracking. 

Among the four types of tracking models (Table 7), the LRCN was applied the most due to reasonable architectures and decent tracking performance. Besides those models, some researchers also came up with some easy but efficient tracking models tailored to animal farming. Object detection models (e.g., faster R-CNN, VGG, YOLO, SSD, FCN, etc.) were utilized to detect and locate animals in images, and then the animals were tracked based on their geometric features in continuous frames using different extra algorithms. These algorithms included popular trackers (e.g., simple online and real-time tracking, SORT; Hungarian algorithm; Munkres variant of the Hungarian assignment algorithm, MVHAA; spatial-aware temporal response filter, STRF; and channel and spatial reliability discriminative correlation filter tracker, CSRDCF) [61,83,88,96,103] and bounding box tracking based on Euclidean distance changes of centroids and probability of detected objects [71,95,118,125,230]. 

## 5. Strategies for Algorithm Development

Appropriate strategies for algorithm development are critical to obtain a robust and efficient model. In one aspect, they need to reduce overfitting in which models may perform well in training data but generalize poorly in testing; in the other aspect, they need to avoid underfitting in which models may perform poorly in both training and testing. In this section, the strategies are organized into distribution of development data, data augmentation, transfer learning, hyperparameter tuning, and evaluation metric. In some model configurations, data augmentation and transfer learning are also categorized with hyperparameters. These two parts have a significant impact on the efficiency of model development, thus being discussed separately. 

### 5.1. Distribution of Development Data 

Reasonable distribution of development data may feed networks with appropriate variations or prevalent patterns in application environments of interest. Data are generally split into training and validation/testing sets (training:validation/testing) or training, validation, and testing sets (training:validation:testing). Validation sets are also called development sets or verification sets and generally used for hyperparameter tuning during model development. In conventional machine learning [231], models are trained with training sets, simultaneously validated with validation sets, and tested with hold-out or unseen testing sets; or if there are limited data, the models are developed with cross-validation strategies, in which a dataset is equally split into several folds, and the models are looped over these folds to determine the optimal model. These strategies are favorable to avoid overfitting and get performance for models facing unseen data. However, strategies of training:validation/testing were adopted more often than training:validation:testing (Figure 8a). Probably because these applications involved a large amounts of data (1000–5000 images in Section 3.5), and the former one is able to learn sufficient variations and produce a robust model. Meanwhile, CNN architectures contain many model parameters to be estimated/updated and hyperparameters to be tuned, and the latter strategy may be time-consuming to train those architectures and thus used infrequently. However, we did recommend the latter strategy, or even cross-validation [11,66,73,90], which may take time yet produce a better model. 

The ratios of 80:20 and 70:30 were commonly used for the strategy of training:validation/testing (Figure 8b). These are also rules of thumb in machine learning [232] to balance variations, training time, and model performance. Training sets with ratios of <50% are generally not recommended since models may not learn sufficient variations from training data and result in poor detection performance. There are some exemptions that if a significant amount of labeled data are available (e.g., 612,000 images in [95] and 356,000 images in [122]), the proportion of training data can be as small as 2–3% due to sufficient variations in these data. Ratios of the strategy of training:validation:testing included 40:10:50, 50:30:20, 60:15:25, 60:20:20, 65:15:20, 70:10:20, 70:15:15, 70:20:10, and 80:10:10 (Figure 8c). Although the frequency of 40:10:50 was as high as that of 70:15:15, it does not mean that 40:10:50 is an optimal ratio. For small datasets, the proportion of training data is expected to be higher than that of testing data because of the abovementioned reasons. 

### 5.2. Data Augmentation 

Data augmentation is a technique to create synthesis images and enlarge limited datasets for training deep learning models, which prevents model overfitting and underfitting. There are many augmentation strategies available. Some of them augment datasets with physical operations (e.g., adding images with high exposure to sunlight) [88]; and some others use deep learning techniques (e.g., adversarial training, neural style transfer, and generative adversarial network) to augment data [233], which require engineering knowledge. However, these are not the focuses in this section because of complexity. Instead, simple and efficient augmentation strategies during training are concentrated [234]. 

Figure 9 shows usage frequency of common augmentation strategies in animal farming. Rotating, flipping, and scaling were three popular geometric transformation strategies. The geometric transformation is to change geometric sizes and shapes of objects. Relevant strategies (e.g., distorting, translating, shifting, reflecting, shearing, etc.) were also utilized in animal farming [55,73]. Cropping is to randomly or sequentially cut small pieces of patches from original images [130]. Adding noises includes blurring images with filters (e.g., median filters [100]), introducing additional signals (e.g., Gaussian noises [100]) onto images, adding random shapes (e.g., dark polygons [70]) onto images, and padding pixels around boundaries of images [100]. Changing color space contains altering components (e.g., hues, saturation, brightness, and contrast) in HSV space or in RGB space [88,216], switching illumination styles (e.g., simulate active infrared illumination [59]), and manipulating pixel intensities by multiplying them with various factors [121]. All these may create variations during training and make models avoid repeatedly learning homogenous patterns and thus reduced overfitting. 

Although data augmentation is a useful technique, there are some sensitive cases that are not suitable for augmentation. Some behavior recognitions may require consistent shapes and sizes of target animals, hence, geometric transformation techniques that are reflecting, rotating, scaling, translating, and shearing are not recommended [66]. Distortions or isolated intensity pixel changes, due to modifying body proportions associated with body condition score (BCS) changes, cannot be applied [108]. Animal face recognition is sensitive to orientations of animal faces, and horizontal flipping may change the orientations and drop classification performance [130].

### 5.3. Transfer Learning 

Transfer learning is to freeze parts of models which contain weights transferred from previous training in other datasets and only train the rest. This strategy can save training time for complex models but does not compromise detection performance. Various similarities between datasets of transfer learning and customized datasets can result in different transfer learning efficiencies [51]. Figure 10 shows popular public datasets for transfer learning in animal science, including PASCAL visual object class dataset (PASCAL VOC dataset), common objects in context (COCO) dataset; motion analysis and re-identification set (MARS), and action recognition dataset (UCF101). Each dataset is specific for different computer vision tasks.

### 5.4. Hyperparameters 

Hyperparameters are properties that govern the entire training process and determine training efficiency and accuracy and robustness of models. They include variables that regulate how networks are trained (e.g., learning rate, optimizer of training) and variables which control network structures (e.g., regularization) [235]. There can be many hyperparameters for specific architectures (e.g., number of region proposals for region-based networks [37], number of anchors for anchor-based networks [173]), but only those generic for any types of architectures or commonly used in animal farming are focused. 

#### 5.4.1. Gradient Descent Mode

One important concept to understand deep learning optimization is gradient descent, in which parameters are updated with gradients in backpropagation of training. There are three major gradient descent algorithms: batch gradient descent, stochastic gradient descent (SGD), and mini-batch SGD [236]. Batch gradient descent is to optimize models based on entire datasets. It may learn every detailed feature in a dataset but be inefficient in terms of time to obtain feedbacks. SGD is to optimize models based on each training sample in a dataset. It may update model fast with noisy data, resulting in a high variance in loss curves. Meanwhile, it is not able to take advantages of vectorization techniques and slow down the training. Mini-batch SGD is to optimize models based on several training samples in a dataset. It may take advantages of batch gradient descent and SGD. In most cases, mini-batch SGD is simplified as SGD. Three hyperparameters determine efficiency of SGD, which are learning rate, batch size, and optimizer of training. It should be noted that batch size is only for SGD or mini-batch SGD. 

#### 5.4.2. Learning Rate 

A learning rate is used to regulate the length of steps during training. Large rates can result in training oscillation and make convergence difficult, while small rates may require many epochs or iterations to achieve convergence and result in suboptimal networks without sufficient epochs or iterations. Nasirahmadi et al. [60] compared learning rates of 0.03, 0.003, and 0.0003, and the middle one performed better. Besides absolute values, modes of learning rate are other factors to consider. Constant learning rates are for maintaining consistent steps throughout the training process [88], and under this mode, initial rates are difficult to decide based on the abovementioned facts. Decay modes may be better options since learning rates are reduced gradually to assist in convergence. One of the modes is linear learning rate decay, in which learning rates are dropped at specific steps (e.g., initially 0.001 for the first 80,000 steps and subsequently 0.0001 for the last 40,000 steps [71]) or dropped iteratively (e.g., initially 0.0003 and dropping by 0.05 every 2 epochs [66]). Another decay mode is exponential learning rate decay, in which learning rates are dropped exponentially (e.g., initially 0.00004 with decay factor 0.95 for every 8000 steps [179]).

#### 5.4.3. Batch Size 

A batch size controls the number of images used in one epoch of training. Large batch sizes may make models learn many variations in one epoch and produce smooth curves but are computationally expensive. The situation is opposite for small batch sizes. Common batch sizes are in a power of 2, ranging from $2^{0}$ to $2^{7}$ in animal farming [91,120]. They depend on memory of devices (e.g., GPU) used for training and input image sizes. Chen et al. [114] compared the batch sizes of 2, 4, 6, and 8 given that number of epochs was controlled at 200, and the batch size of 2 had slightly better performance than the others. Perhaps, larger batch sizes also needed a greater number of epochs to get convergence. Another influential factor is complexity of architectures. Arago et al. [91] deployed batch sizes of 1 for faster R-CNN and 4 for SSD. Perhaps, complex architectures with small batch sizes can speed up training, while simple and lightweight large batch sizes can improve training efficiency. 

#### 5.4.4. Optimizer of Training 

An optimizer is to minimize the training loss during training and influences stability, speed, and final loss of convergence. Common optimizers are Vanilla SGD [237], SGD with momentum (typically 0.9) [238], SGD with running average of its recent magnitude (RMSProp) [239], and SGD with adaptive moment estimation (Adam) [240]. Vanilla SGD suffers from oscillation of learning curves and local minima, which slows down the training speed and influences convergence. SGD with momentum is to multiply gradient with a moving average. It, as indicated by term “momentum”, speeds up in the directions with gradients, damps oscillations in the directions of high curvature, and avoids local minima. SGD with RMSProp is to divide gradient with a moving average. It alleviates variances of magnitude of gradient among different weights and allows a larger learning rate, which improves the training efficiency. SGD with Adam is to combine momentum method and RMSProp method and owns the strengths of both. However, it does not mean that the Adam method is the only solution for algorithm optimization. Based on the investigation, there were 4 publications for Vanilla SGD, 16 for SGD with momentum, 2 for SGD with RMSProp, and 7 for SGD with Adam. When Wang et al. [156] tested these optimizers, Vanilla SGD even outperformed the others if it was appropriately regularized. Adadelta and Ranger, which were the other two efficient optimizers and outperformed Vanilla SGD [241,242], were also applied in animal farming [70,84]. 

#### 5.4.5. Regularization 

Regularization is to place penalty onto training, so that models do not match well for training and can generalize well in different situations. Dropout is one of the efficient regularization methods to control overfitting and randomly dropped units (along with their connections) from network during training [243]. It should be noted that a high dropout ratio can decrease the model performance. Wang et al. [156] compared dropout ratios of 0.3, 0.5, and 0.7, and the 0.7 ratio decreased over 20% of performance compared to the 0.3 ratio. Dropping too many units may decrease the model capacity to handle variations and lead to poor performance, while dropping too few units may not regularize models well. A ratio of 0.5 may address the dilemma [74]. Another regularization method used in animal farming is batch normalization [47,194]. It normalizes parameters in each layer with certain means and variance, which maintains parameter distribution constant, reduces gradient dispersion, and makes training robust [244]. Other regularization methods (e.g., L1/L2 regularization), which add L1/L2 norms to loss function and regularize parameters, were also utilized in animal farming [76,111]. 

#### 5.4.6. Hyperparameters Tuning 

Training a deep learning model may involve many hyperparameters, and each hyperparameter has various levels of values. Choosing an optimal set of hyperparameters for tuning models is difficult. Furthermore, as the “no-free-lunch” theorem suggests that there is no algorithm that can outperform all others for all problems [245]. An optimal set of hyperparameters may work well on particular datasets and models but perform differently when situations change. Therefore, hyperparameters also need to be tuned to get optimal ones in particular situations. Considering the amount of hyperparameters, conventional hyperparameter searching methods for machine learning (e.g., random layout, grid layout [235]) may be time-consuming. There are better methods to optimize the values of hyperparameters. Alameer et al. [88] selected an optimal set of hyperparameters using nested-cross validation in an independent dataset and then trained models with the optimal values. Nested-cross validation, like cross validation, uses *k − 1* folds of data for training and the rest for testing to select optimal values of hyperparameters [246]. Salama et al. [55] iteratively searched optimal values of hyperparameters using the Bayesian optimization approach, which is to jointly optimize a group of values based on probability [247].

### 5.5. Evaluation Metrics 

Evaluation should be conducted during training or testing to understand the correctness of model prediction. For evaluation during training, investigators can observe model performance in real time and adjust training strategies timely if something is wrong; and as for evaluation during testing, it can provide final performance of trained models. Different evaluation metrics can assist researchers in evaluating models from different perspectives. Even with the same metric, different judgment confidences can result in different performance [101]. Different loss functions are generally used to evaluate the errors between ground truth and prediction during training. These include losses for classification, detection, and segmentation [51,111]. Accuracy over different steps is also applied during training [126]. Although evaluation metrics during training can help optimize models, final performance of models during testing is of major concern.

Common metrics to evaluate CNN architectures during testing are presented in Table 8. Except for false negative rate, false positive rate, mean absolute error, mean square error, root mean square error, and average distance error, higher values of the rest metrics indicate better performance of models. Among these metrics, some (e.g., accuracy, precision, recall, etc.) are generic for multiple computer vision tasks, but some (e.g., Top-5 accuracy for image classification, panoptic quality for segmentation, overlap over time for tracking, etc.) are specific for single computer vision tasks. Generic evaluation metrics account for the largest proportion among the metrics. Some focus on object presence (e.g., average precision), some focus on object absence (e.g., specificity), and some focus on both (e.g., accuracy). Among object presence prediction, misidentification (wrongly recognizing others as target objects) and miss-identification (wrongly recognizing target objects as none) are the common targets of concern. Regression metrics (e.g., mean absolute error, mean square error, root mean square error, etc.) are used to evaluate the difference of continuous variables between ground truth and prediction. Besides a single value, some metrics (e.g., precision-recall curve) have multiple values based on different confidences and are plotted as curves. Processing speed, a critical metric for real-time application, is typically used to evaluate how fast a model can process an image.

Existent metrics sometimes may not depict the performance of interest in some applications, and researchers need to combine multiple metrics. Seo et al. [89] integrated average precision and processing speed to comprehensively evaluate the models based on the tradeoff of these two metrics. They also combined these two metrics with device price and judged which models were more computationally and economically efficient. Fang et al. [75] proposed a mixed tracking metric by combining overlapping area, failed detection, and object size.

## 6. Performance

### 6.1. Performance Judgment

Model performance should be updated until it meets the expected acceptance criterion. Maximum values of evaluation metrics in the 105 publications are summarized in Figure 11. These generic metrics included accuracy, specificity, recall, precision, average precision, mean average precision, F1 score, and intersection over union. They were selected because they are commonly representative as described in Table 8. One publication can have multiple evaluation metrics, and one evaluation metric can have multiple values. Maximum values were selected since they significantly influenced whether model performance was acceptable and whether the model was chosen. Most researchers accepted performance of over 90%. The maximum value can be as high as 100% [69] and as low as 34.7% [92]. Extremely high performance may be caused by overfitting, and extremely low performance may be resulted from underfitting or lightweight models. Therefore, these need to be double-checked, and models need to be developed with improved strategies. 

### 6.2. Architecture Performance 

Performance can vary across different architectures and is summarized in this section. Due to large inconsistencies in evaluation metrics for object detection, semantic/instance segmentation, pose estimation, and tracking, only the accuracy for image classification of animal farming was organized in Table 9. There could be multiple values of accuracy in one paper, and only the highest values were obtained. Inconsistent performance appeared in the tested references due to varying datasets, development strategies, animal species, image quality, etc. Some lightweight architectures (e.g., MobileNet) can even outperform the complicated architectures (e.g., NASNet) with proper training (Table 9). Some architectures (e.g., AlexNet, LetNet5, and VGG19) had a wide range (over 30% difference) of performance in animal farming, likely due to various levels of difficulties in the tasks. General performance (i.e., Top-1 accuracy) of these models was also extracted from the benchmark dataset of image classification (ImageNet [250]) for the reference of architecture selection of future applications. Most accuracies in animal farming were much higher than those in ImageNet with the same models, which can be attributed to fewer object categories and number of instances per image in the computer vision tasks of animal farming.

## 7. Applications

With developed CNN-based computer vision systems, their applications were summarized based on year, country, animal species, and purpose (Figure 12). It should be noted that one publication can include multiple countries and animal species.

### 7.1. Number of Publications Based on Years 

The first publication of CNN-based computer vision systems in animal farming appeared in 2015 (Figure 12a) [249], which coincided with the onset of rapid growth of modern CNNs in computer science as indicated in Figure 2. Next, 2016 and 2017 yielded little development. However, the number of publications grew tremendously in 2018, maintained at the same level in 2019, and doubled by October 2020. It is expected that there will be more relevant applications ongoing. A delay in the use of CNN techniques in animal farming has been observed. Despite open-source deep learning frameworks and codes, direct adoption of CNN techniques into animal farming is still technically challenging for agricultural engineers and animal scientists. Relevant educational and training programs are expected to improve the situation, so that domain experts can gain adequate knowledge and skills of CNN techniques to solve emerging problems in animal production. 

### 7.2. Number of Publications Based on Countries 

China has applied CNN techniques in animal farming much more than other countries (Figure 12b). According to the 2020 report from USDA Foreign Agricultural Service [251], China had 91,380,000 cattle ranking number 4 in the world, 310,410,000 pigs ranking number 1, and 14,850,000 metric tons of chickens ranking number 2. It is logical that precision tools are needed to assist farm management in such intensive production. Developed countries, such as USA, UK, Australia, Belgium, and Germany, also conducted CNN research, since the techniques are needed to support intensive animal production with less labor in these countries [252]. It should be pointed out that only papers published in English were included in this review, as the language can be understood by all authors. Excluding papers in other languages, such as Chinese, French, and German, could have altered the results of this review investigation.

### 7.3. Number of Publications Based on Animal Species 

Cattle and pig are two animal species that were examined the most (Figure 12c). Although cattle, pig, and poultry are three major sources of meat protein [251], poultry are generally reared in a higher density than the other two (10–15 m^2^/cow for cattle in a freestall system [253], 0.8–1.2 m^2^/pig for growing-finishing pigs [254], and 0.054–0.07 m^2^/bird for broilers raised on floor [255]). High stocking density in poultry production leads to much occlusion and overlapping among birds, and low light intensity can cause low-resolution images [13]. These make automated detection in poultry production challenging. Alternatively, researchers may have trials of CNN techniques in relatively simple conditions for cattle and pigs. Sheep/goat are generally reared in pasture, where cameras may not be able to cover every sheep/goat [65], which is an obstacle to develop CNN-based computer vision systems for sheep/goat production.

### 7.4. Number of Publications Based on Purposes 

Purposes of system applications are categorized into abnormality detection, animal recognition, behavior recognition, individual identification, and scoring (Figure 12d). Abnormality detection can signal early warnings for farmers and contribute to timely intervention and mitigation of the problems. Abnormality includes pain reflected by face expression [76], lameness [152], mastitis [53], and sickness [78]. Animal recognition is to recognize animals of interest from background and generally used for animal counting, so that usage status of facilities can be understood [230]. Behavior recognition is to detect different behaviors of animals, such as feeding, drinking, mounting, stretching, etc. [51,86,90,119]. Behaviors of animals reflect their welfare status and facility utilization efficiency, as well as signs of abnormality. Individual identification is to identify individual animals based on the features from back of head [111], faces [49], body coatings [100], muzzle point [116], and additional code [54]. By identification, production performance, health status, and physiological response of individuals may be linked together and tracked through the entire rearing process. Scoring is to estimate the body conditions of animals, which were typically associated with production, reproduction, and health of animals [101]. 

Animal recognition, behavior recognition, and individual identification occupy large proportions in the five categories of purposes (Figure 12d). Based on the above definition, one category can achieve one or two functions of others. However, abnormality detection and scoring, per the best knowledge of the authors, require veterinary or animal science expertise, which is challenging for some engineers of system development. Therefore, computer science researchers may prioritize the three categories for exploring the potential of CNN techniques in animal farming.

## 8. Brief Discussion and Future Directions

In this section, emerging problems from the review are briefly discussed and some directions of future research are provided for CNN-based computer vision systems in animal farming.

### 8.1. Movable Solutions 

Conventional computer vision systems require installation of fixed cameras in optimal locations (e.g., ceiling, passageway, gate, etc.) to monitor the areas of interest. To cover an entire house or facility, multiple cameras are utilized and installed in strategic locations [6]. This method relies on continual and preventative maintenance of multiple cameras to ensure sufficient information and imaging of the space. An alternative method is to use movable computer vision systems. Chen et al. [61] placed a camera onto an inspection robot capable of navigating along a fixed rail system near the ceiling of a pig house. The UAVs were also used to monitor cattle and/or sheep in pastures and may potentially be used inside animal houses and facilities [45,57,120]. The UAVs move freely in air space without occlusion by animals and may serve to assist farmers in managing animals. But they are difficult to navigate inside of structures due to obstacles and loss of Global Positioning System signal, and their efficiency is still needed to be verified. To achieve real-time movable inspection, some lightweight models (e.g., SSD, YOLO) of computer vision systems are recommended for monitoring farm animals.

### 8.2. Availability of Datasets 

Current CNN techniques require adequate data to learn data representation of interest. In this regard, available datasets and corresponding annotations are critical for model development. Publicly-available datasets contain generic images (e.g., cars, birds, pedestrians, etc.) for model development. However, few datasets with annotations for animal farming are available (Table 10). Lack of availability of animal farming datasets is due to confidentiality of data and protocols within animal production research and industries. Although transfer learning techniques aid in transferring pre-trained weights of models from publicly available datasets into customized datasets, which lessens model development time, high similarities between those datasets may improve transfer learning efficiency and model performance [51]. Environments for capturing generic images are quite different from those encountered in animal farming, which may downgrade transfer learning. Therefore, animal-farming-related datasets are urgently needed for engineers or scientists to develop models tailored to animal farming.

### 8.3. Research Focuses

Some items were found in smaller proportions compared with their counterparts in animal farming, such as fewer CNN architectures of pose estimation out of the five computer vision tasks, fewer applications of poultry out of the four farm animal species, and fewer applications of abnormality detection and scoring out of the five purposes. These might be caused by complexity of detected objects, poor environments for detection, and limited expertise knowledge. These areas may need more research focuses than their counterparts to fill the research and knowledge gaps. 

### 8.4. Multidisciplinary Cooperation 

Models are always among central concern for developing robust computer vision systems. Despite many advanced CNN architectures being applied to animal farming, more cutting-edge and better models (e.g., YOLO V5) have not been introduced into the animal industry. Alternatively, there were only a few studies exploring the useful information for animal farming using trained CNN architectures, which included animal behavior performance with food restriction [66,88], lighting conditions [11], rearing facilities [85], and time of a day [85,122]. Most studies primarily focused on evaluating model performance, from which animal management strategies were still not improved, due to limitations of knowledge, time, and resources from single disciplines. Enriched research and applications of CNN-based computer vision systems require multidisciplinary collaboration to synergize expertise across Agricultural Engineering, Computer Science, and Animal Science fields to produce enhanced vision systems and improve precision management strategies for the livestock and poultry industries.

## 9. Conclusions

In this review, CNN-based computer vision systems were systematically investigated and reviewed to summarize the current practices and applications of the systems in animal farming. In most studies, the vision systems performed well with appropriate preparations, choices of CNN architectures, and strategies of algorithm development. Preparations influence quality of data fed into models and included camera settings, inclusion of variations for data recordings, GPU selection, image preprocessing, and data labeling. Choices of CNN architectures may be based on tradeoffs between detection accuracy and processing speed, and architectures vary with respect to computer vision tasks. Strategies of algorithm development involve distribution of development data, data augmentation, transfer learning, hyperparameter tuning, and choices of evaluation metrics. Judgment of model performance and performance based on architectures were presented. Applications of CNN-based systems were also summarized based on year, country, animal species, and purpose.

Four directions of CNN-based computer vision systems in animal farming were provided. (1) Movable vision systems with lightweight CNN architectures can be alternative solutions to inspect animals flexibly and assist in animal management. (2) Farm-animal-related datasets are urgently needed for tailored model development. (3) CNN architectures for pose estimation, poultry application, and applications of abnormality detection and scoring should draw more attention to fill research gaps. (4) Multidisciplinary collaboration is encouraged to develop cutting-edge systems and improve management strategies for livestock and poultry industries. 

## Figures and Tables

**Table 1:** Specifications and approximate price of GPU cards and corresponding references.

GPU	# of CUDA Cores	FPP (TFLOPS)	MMB (GB/s)	Approximate Price ($)	Reference
NVIDIA GeForce GTX Series					
970	1664	3.4	224	157	[65,66]
980 TI	2816	5.6	337	250	[52,67,68]
1050	640	1.7	112	140	[69,70]
1050 TI	768	2.0	112	157	[71,72,73]
1060	1280	3.9	121	160	[60,74,75]
1070	1920	5.8	256	300	[76,77]
1070 TI	2432	8.2	256	256	[78]
1080	2560	8.2	320	380	[79,80]
1080 TI	3584	10.6	484	748	[81,82,83], etc.
1660 TI	1536	5.4	288	290	[84]
TITAN X	3072	6.1	337	1150	[45,59]
NVIDIA GeForce RTX Series					
2080	4352	10.6	448	1092	[85,86,87], etc.
2080 TI	4352	14.2	616	1099	[42,88,89], etc.
TITAN	4608	16.3	672	2499	[47,90]
NVIDIA Tesla Series					
C2075	448	1.0	144	332	[43]
K20	2496	3.5	208	200	[91]
K40	2880	4.3	288	435	[92,93]
K80	4992	5.6	480	200	[94,95]
P100	3584	9.3	732	5899	[96,97,98]
NVIDIA Quadro Series					
P2000	1024	2.3	140	569	[99]
P5000	2560	8.9	288	800	[53]
NVIDIA Jetson Series					
NANO	128	0.4	26	100	[89]
TK1	192	0.5	6	60	[100]
TX2	256	1.3	60	400	[89]
Others					
NVIDIA TITAN XP	3840	12.2	548	1467	[101,102,103]
Cloud server	—	—	—	—	[54,104]
CPU only	—	—	—	—	[64,105,106], etc.

**Note:** GPU is graphics processing unit; CPU is central processing unit; CUDA is computer unified device architecture; FPP is floating-point performance; TFLOPS is tera floating point operation per second; and MMB is maximum memory bandwidth. “—” indicates missing information.

**Table 2:** Available labeling tools for different computer vision tasks.

Computer Vision Task	Tool	Source	Reference
Object detection	LabelImg	GitHub [133] (Windows version)	[71,91,134], etc.
	Image Labeler	MathWorks [135]	[131]
	Sloth	GitHub [136]	[113]
	VATIC	Columbia Engineering [137]	[130]
Semantic/instance segmentation	Graphic	Apple Store [138]	[92]
	Supervisely	SUPERVISELY [139]	[104]
	LabelMe	GitHub [140]	[64,90,112]
	VIA	Oxford [141]	[46,51]
Pose estimation	DeepPoseKit	GitHub [142]	[97]
	DeepLabCut	Mathis Lab [143]	[132]
Tracking	KLT tracker	GitHub [144]	[88]
	Interact Software	Mangold [145]	[72]
	Video Labeler	MathWorks [146]	[68]

**Note:** VATIC is Video Annotation Tool from Irvine, California; VIA is VGG Image Annotator; and KLT is Kanade-Lucas-Tomasi.

**Table 3:** Convolutional neural network architectures for image classification.

Model	Highlight	Source (Framework)	Reference
Early versions of CNN			
AlexNet [33]	Classification error of 15.3% in ImageNet	GitHub [154] (TensorFlow)	[55,76,131]
LeNet5 [27]	First proposal of modern CNN	GitHub [155] (PyTorch)	[156]
Inception family			
Inception V1/GoogLeNet [35]	Increasing width of networks, low computational cost	GitHub [157] (PyTorch)	[66,76]
Inception V3 [158]	Inception module, factorized convolution, aggressive regularization	GitHub [159] (TensorFlow)	[63,76,120]
Inception ResNet V2 [160]	Combination of Inception module and Residual connection	GitHub [161] (TensorFlow)	[69,120]
Xception [162]	Extreme inception module, depthwise separable convolution	GitHub [163] (TensorFlow)	[120]
MobileNet family			
MobileNet [149]	Depthwise separable convolution, lightweight	GitHub [159] (TensorFlow)	[120]
MobileNet V2 [164]	Inverted residual structure, bottleneck block	GitHub [165] (PyTorch)	[120]
NASNet family			
NASNet Mobile [150]	Convolutional cell, learning transformable architecture	GitHub [166] (TensorFlow)	[120]
NASNet Large [150]		GitHub [159] (TensorFlow)	[120]
Shortcut connection networks			
DenseNet121 [39]	Each layer connected to every other layer, feature reuse	GitHub [167] (Caffe, PyTorch, TensorFlow, Theano, MXNet)	[120,151]
DenseNet169 [39]			[120]
DenseNet201 [39]			[69,76,120]
ResNet50 [36]	Residual network, reduction of feature vanishing in deep networks	GitHub [168] (Caffe)	[69,76,151], etc.
ResNet101 [36]			[120]
ResNet152 [36]			[120]
VGGNet family			
VGG16 [34]	Increasing depth of networks	GitHub [169] (TensorFlow)	[49,107,151], etc.
VGG19 [34]			[120,131]
YOLO family			
YOLO [148]	Regression, fast network (45 fps)	GitHub [170] (Darknet)	[74]
DarkNet19 [171]	Fast, accurate YOLO-based network	GitHub [172] (Chainer)	[76]

**Note:** “Net” in model names is network, and number in model names is number of layers of network. AlexNet is network designed by Alex Krizhevsky; CNN is convolutional neural network; DenseNet is densely connected convolutional network; GoogLeNet is network designed by Google Company; LeNet is network designed by Yann LeCun; NASNet is neural architecture search network; ResNet is residual network; VGG is visual geometry group; Xception is extreme inception network; and YOLO is you only look once.

**Table 4:** Convolutional neural network architectures for object detection.

Model	Highlight	Source (Framework)	Reference
Fast detection networks			
RFBNetSSD [174]	RFB, high-speed, single-stage, eccentricity	GitHub [177] (PyTorch)	[44]
SSD [173]	Default box, box adjustment, multi-scale feature maps	GitHub [178] (Caffe)	[78,134,179], etc.
YOLO9000 [171]	9000 object categories, YOLO V2, joint training	GitHub [180] (Darknet)	[105,128]
YOLO V2 [171]	K-mean clustering, DarkNet-19, multi-scale	GitHub [181] (TensorFlow)	[45,89,100], etc.
Tiny YOLO V2 [175]		GitHub [182] (TensorFlow)	[89]
YOLO V3 [175]	Logistic regression, logistic classifier, DarkNet-53, skip-layer concatenation	GitHub [183] (PyTorch)	[85,99,102], etc.
YOLO V4 [176]	WRC, CSP, CmBN, SAT, Mish-activation	GitHub [184] (Darknet)	[71]
Region-based networks			
R-CNN [185]	2000 region proposals, SVM classifier	GitHub [186] (Caffe)	[56,110]
Faster R-CNN [37]	RPN, fast R-CNN, sharing feature maps	GitHub [187] (TensorFlow)	[81,94,99], etc.
Mask R-CNN [38]	Instance segmentation, faster R-CNN, FCN, ROIAlign	GitHub [188] (TensorFlow)	[51,64,106]
R-FCN [189]	Position-sensitive score map, average voting, shared FCN	GitHub [190] (MXNet)	[60]
Shortcut connection networks			
DenseNet [39]	Each layer connected to every other layer, feature reuse	GitHub [167] (Caffe, PyTorch, TensorFlow, Theano, MXNet)	[115]
ResNet50 [36]	Residual network, reduction of feature vanishing in deep networks	GitHub [168] (Caffe)	[191]
ResNeXt [192]	Cardinality, same topology, residual network, expanding network width	GitHub [193] (Torch)	[194]

**Note:** “Net” in model names is network, and number in model names is number of layers of network. CmBN is cross mini-batch normalization; CSP is cross stage partial connection; ResNet is residual network; ResNext is residual network with next dimension; RFB is receptive filed block; R-CNN is region-based convolutional neural network; R-FCN is region-based fully connected network; ROIAlign is region of interest alignment; RPN is region proposal network; SAT is self-adversarial training; SSD is single shot multibox detector; SVM is support vector machine; WRC is weighted residual connection; and YOLO is you only look once.

**Table 5:** Convolutional neural network architectures for semantic/instance segmentation.

Model	Highlight	Source (Framework)	Reference
Semantic segmentation networks			
DeepLab [200]	Atrous convolution, field of view, ASPP, fully-connected CRF, sampling rate	Bitbucket [203] (Caffe)	[62,105,111]
ERFNet [201]	Residual connection, factorized convolution, high speed with remarkable accuracy, 83 fps	GitHub [204] (PyTorch)	[112]
FCIS [199]	Position-sensitive inside/outside score map, object classification, and instance segmentation jointly	GitHub [205] (MXNet)	[93]
FCN8s [197]	Classification networks as backbones, fully convolutional network, 8-pixel stride	GitHub [206] (PyTorch)	[93]
UNet [198]	Data augmentation, contrasting path, symmetric expanding path, few images for training	GitHub [207] (PyTorch)	[50,59]
Instance segmentation networks			
Mask R-CNN [38]	faster R-CNN, object detection, parallel inference, FCN, ROIAlign	GitHub [188] (TensorFlow)	[93,127,208], etc.
Mask Scoring R-CNN [202]	Mask IOU network, mask quality, mask R-CNN	GitHub [209] (PyTorch)	[46]

**Note:** ASPP is atrous spatial pyramid pooling; Atrous is algorithm *à* trous (French) or hole algorithm; CRF is conditional random field; ERFNet is efficient residual factorized network; FCIS is fully convolutional instance-aware semantic segmentation; FCN is fully convolutional network; IOU is intersection over union; R-CNN is region-based convolutional neural network; ROIAlign is region of interest alignment; and UNet is a network in a U shape.

**Table 6:** Convolutional neural network architectures for pose estimation.

Model	Highlight	Source Code (Framework)	Reference
Heatmap-based networks			
CPHR [212]	Detection heatmap, regression on heatmap, cascade network	GitHub [215] (Torch)	[216]
CPMs [213]	Sequential network, natural learning objective function, belief heatmap, multiple stages and views	GitHub [217] (Caffe, Python, Matlab)	[216]
Hourglass [214]	Cascaded network, hourglass module, residual connection, heatmap	GitHub [218] (Torch)	[97,104,216], etc.
Heatmap-free networks			
DeepLabCut [211]	ROI, residual network, readout layer	GitHub [219] (Python, C++)	[132]
DeepPose [210]	Holistic fashion, cascade regressor, refining regressor	GitHub [220] (Chainer)	[132]

**Note:** CPHR is convolutional part heatmap regression; CPMs is convolutional pose machines; and ROI is region of interest.

**Table 7:** Convolutional neural network architectures for tracking.

Model	Highlight	Source Code (Framework)	Reference
GOTURN [223]	100 fps, feed-forward network, object motion and appearance	GitHub [224] (C++)	[75]
SlowFast network [225]	Low and high frame rates, slow and high pathways, lightweight network	GitHub [226] (PyTorch)	[47]
Two-stream CNN [221]	Complementary information on appearance, motion between frames,	GitHub [227] (Python)	[80]
(Inception, ResNet, VGG, and Xception) with LSTM [222]	Recurrent convolution, CNN, doubly deep in spatial and temporal layers, LSTM	GitHub [228] (PyTorch) GitHub [229] (PredNet)	[86,87,119], etc.

**Note:** CNN is convolutional neural network; GOTRUN is generic object tracking using regression networks; LSTM is long short-term memory; ResNet is residual network; and VGG is visual geometry group.

**Table 8:** Metrics for evaluating performance of convolutional neural network architectures.

Metric	Equation	Brief Explanation	Reference
Generic metrics for classification, detection, and segmentation			
Accuracy	$\frac{TP+TN}{TP+FP+TN+FN}$	Commonly used, comprehensive evaluation of predicting object presence and absence.	[51,101,108], etc.
AP	$\frac{1}{n}\sum_{i=1}^{n}P_{interp}\left(i\right)$	Average performance of misidentification of object presence for a single class. $P_{interp}\left(i\right)$ is the *i*th interpolated precision over a precision-recall curve.	[51,191]
AP@0.5, AP@0.7, AP@0.75, AP@0.5:0.95	—	COCO, evaluation of predicting object presence with different confidence (IOU: >0.5, >0.7, >0.75, and 0.5 to 0.95 with step 0.05)	[72,92,191]
AUC	—	Comprehensive evaluation of miss-identification and misidentification of object presence	[45,248]
Cohen’s Kappa	$\frac{P_{o}-P_{e}}{1-P_{e}},\;P_{o}=accuracy,\;P_{e}=P_{yes}+P_{no}$ $P_{yes}=\frac{TP+FP}{Total}\times \frac{TP+FN}{Total},$ $P_{no}=\frac{FN+TN}{Total}\times \frac{FP+TN}{Total}$	Comprehensive evaluation of classification based on confusion matrix	[76,249]
Confusion matrix	—	Table presentation of summarization of correct and incorrect prediction	[66,101,111], etc.
False negative rate	$\frac{FN}{FN+TP}$	Evaluation of incorrect recognition of object absence	[49,87]
False positive rate	$\frac{FP}{FP+TN}$	Evaluation of incorrect recognition of object presence	[70,73,87], etc.
F1 score	$\frac{2\times Precision\times Recall}{Precision+Recall}$	Comprehensive evaluation of predicting object presence	[51,101,108], etc.
IOU	$\frac{Overlapping\;area}{Union\;area}$	Evaluation of deviation between ground truth area and predicted area	[88,111]
MCC	$\frac{TP\times TN-FP\times FN}{\sqrt{\left(TP+FN\right)\left(TP+FP\right)\left(TN+FP\right)\left(TN+FN\right)}}$	Evaluation of difference between correct prediction and incorrect prediction for object presence and absence	[90]
Mean AP	$\frac{1}{n}\sum_{i=1}^{n}AP\left(i\right)$	Comprehensive evaluation of predicting presence of multiple classes. AP(i) is AP of the *i*th class.	[88,96,102], etc.
Recall/sensitivity	$\frac{TP}{TP+FN}$	Evaluation of miss-identification of object presence	[51,101,108], etc.
Precision	$\frac{TP}{TP+FP}$	Evaluation of misidentification of object presence	[51,101,108], etc.
Specificity	$\frac{TN}{FP+TN}$	Evaluation of predicting object absence	[86,114,119], etc.
Processing speed	$\frac{Number\;of\;images}{Processing\;time}$	Evaluation of speed processing images	[51,81,128], etc.
Generic metrics for regression			
Coefficient of determination (R^2^)	$1-\frac{\sum_{i=1}^{n}\left(y_{i}-\bar{y}\right)}{\sum_{i=1}^{n}\left(y_{i}-{\hat{y}}_{i}\right)}$	Comprehensive evaluation of prediction errors based on a fitted curve. $y_{i}$ is the *i*th ground truth values, ${\hat{y}}_{i}$ is the *i*th predicted value, and $\bar{y}$ is average of *n* data points	[50,115,121]
Mean absolute error	$\frac{1}{n}\sum_{i=1}^{n}\left|y_{i}-{\hat{y}}_{i}\right|$	Evaluation of absolute deviation between ground truth values ($y_{i}$) and predicted values (${\hat{y}}_{i}$) over *n* data points	[54,194]
Mean square error	$\frac{1}{n}\sum_{i=1}^{n}{\left(y_{i}-{\hat{y}}_{i}\right)}^{2}$	Evaluation of squared deviation between ground truth values ($y_{i}$) and predicted values (${\hat{y}}_{i}$) over *n* data points	[54,73,96], etc.
RMSE	$\sqrt{\frac{1}{n}\sum_{i=1}^{n}{\left(y_{i}-{\hat{y}}_{i}\right)}^{2}}$	Evaluation of root-mean-squared deviation between ground truth values ($y_{i}$) and predicted values (${\hat{y}}_{i}$) over *n* data points	[73,194]
Generic metrics with curves			
F1 score-IOU curve	—	Comprehensive evaluation of miss-identification and misidentification of object presence based on different confidence	[64,106]
Recall-IOU curve	—	Evaluation of miss-identification of object presence based on different confidence	[64,106]
Precision-IOU curve	—	Evaluation of misidentification of object presence based on different confidence	[64,106]
Precision-recall curve	—	Evaluation of misidentification of object presence based on number of detected objects	[45,99,196], etc.
Specific metrics for image classification			
Top-1, Top-3, and Top-5 accuracy	—	ImageNet, evaluation of whether a target class is the prediction with the highest probability, top 3 probabilities, and top 5 probabilities.	[47,63]
Specific metrics for semantic/instance segmentation			
Average distance error	$\frac{A_{union}-A_{overlap}}{T_{contour}}$	Comprehensive evaluation of segmentation areas and segmentation contours. $A_{union}$ is union area; $A_{overlap}$ is overlapping area; and $T_{contour}$ is perimeter of extracted contour.	[127]
Mean pixel accuracy	$\frac{1}{k+1}\sum_{i=0}^{k}\frac{P_{ii}}{\sum_{j=0}^{k}P_{ij}}$	Comprehensive evaluation of segmenting multiple classes. k is total number of classes expect for background; $P_{ii}$ is total number of true pixels for class *i*; $P_{ij}$ is total number of predicted pixels for class *i*.	[90,127]
Panoptic quality	$\frac{\sum_{s\in TP}IOU}{TP+\frac{1}{2}FP+\frac{1}{2}FN}$	Comprehensive evaluation of miss-identified and misidentified segments. *s* is segment.	[59]
Specific metrics for pose estimation			
PCKh	$\frac{\sum_{i=1}^{n}\delta \left(\|P_{i,\;j}-T_{ij}\|-threshold\times head\_size_{i}\right)}{n}$	Evaluation of correctly detected key points based on sizes of object heads. *n* is number of images; $P_{i,\;j}$ is the *j*th predicted key points in the *i*th image; $T_{ij}\;$is the *j*th key points of ground truth in the *i*th image; and $head\_size_{i}$ is the length of heads in the *i*th image	[216]
PDJ	—	Evaluation of correctly detected parts of objects	[210]
Specific metrics for tracking			
MOTA	$1-\frac{\sum_{t}FN_{t}+FP_{t}+{\left(ID\;switch\right)}_{t}}{\sum_{t}GT_{t}}$	Evaluation of correctly tracking objects over time. *t* is the time index of frames; and *GT* is ground truth.	[83,88,96]
MOTP	$\frac{\sum_{i,\;t}d_{t}^{i}}{\sum_{t}c_{t}}$	Evaluation of location of tracking objects over time. *t* is time index of frames; *i* is index of tracked objects; *d* is distance between target and ground truth; and *c* is number of ground truth	[88]
OTP	$\frac{\sum_{MTU_{i}}Boxes_{i,f=30}}{\sum_{MTU_{i}}Boxes_{i,f=1}}$	Evaluation of tracking objects in minimum tracking units (MTU). $Boxes_{i,f=30}$ is the number of bounding boxes in the first frame of the *i*th MTU; $Boxes_{i,f=1}$ is the number of bounding boxes in the last frame of the *i*th MTU.	[72]
Overlap over time	—	Evaluation of length of objects that are continuously tracked	[75]

**Note:** AP is average precision; AUC is area under curve; COCO is common objects in context; FP is false positive; FN is false negative; IOU is intersection over union; MCC is Matthews correlation coefficient; MOTA is multi-object tracker accuracy; MOTP is multi-object tracker precision; OTP is object tracking precision; PDJ is percentage of detected joints; PCKh is percentage of correct key points according to head size; RMSE is root mean square error; TP is true positive; and TN is true negative. “—” indicates missing information.

**Table 9:** Accuracy of architectures for image classification.

Model	Accuracy in Animal Farming (%)	Top-1 Accuracy in ImageNet (%)	Reference
Early versions of CNN			
AlexNet [33]	60.9–97.5	63.3	[55,76,131]
LeNet5 [27]	68.5–97.6	−	[156]
Inception family			
Inception V1/GoogLeNet [35]	96.3–99.4	−	[66,76]
Inception V3 [158]	92.0–97.9	78.8	[63,76,120]
Inception ResNet V2 [160]	98.3–99.2	80.1	[69,120]
Xception [162]	96.9	79.0	[120]
MobileNet family			
MobileNet [149]	98.3	−	[120]
MobileNet V2 [164]	78.7	74.7	[120]
NASNet family			
NASNet Mobile [150]	85.7	82.7	[120]
NASNet Large [150]	99.2	−	[120]
Shortcut connection networks			
DenseNet121 [39]	75.4–85.2	75.0	[120,151]
DenseNet169 [39]	93.5	76.2	[120]
DenseNet201 [39]	93.5–99.7	77.9	[69,76,120]
ResNet50 [36]	85.4–99.6	78.3	[69,76,151], etc.
ResNet101 [36]	98.3	78.3	[120]
ResNet152 [36]	96.7	78.9	[120]
VGGNet family			
VGG16 [34]	91.0–100	74.4	[49,107,151], etc.
VGG19 [34]	65.2–97.3	74.5	[120,131]
YOLO family			
YOLO [148]	98.4	−	[74]
DarkNet19 [171]	95.7	−	[76]

**Note:** “Net” in model names is network, and number in model names is number of layers of network. AlexNet is network designed by Alex Krizhevsky; CNN is convolutional neural network; DenseNet is densely connected convolutional network; GoogLeNet is network designed by Google Company; LeNet is network designed by Yann LeCun; NASNet is neural architecture search network; ResNet is residual network; VGG is visual geometry group; Xception is extreme inception network; and YOLO is you only look once. “−” indicates missing information.

**Table 10:** Available datasets of animal farming.

Computer Vision Task	Name of Dataset	Animal Species	Size of Images	# of Images	Annotation (Y/N)	Source	Reference
Image classification	AerialCattle2017	Cattle	681 × 437	46,340	N	University of BRISTOL [256]	[57]
	FriesianCattle2017	Cattle	1486 × 1230	840	N		[57]
Object detection	Aerial Livestock Dataset	Cattle	3840 × 2160	89	N	GitHub [257]	[196]
	Pigs Counting	Pig	200 × 150	3401	Y	GitHub [258]	[194]
	—	Cattle	4000 × 3000	670	Y	Naemura Lab [259]	[45]
	—	Pig	1280 × 720	305	Y	UNIVERSITAT HOHENHEIM [260]	[113]
	—	Poultry	412 × 412	1800	N	Google Drive [261]	[126]
Pose estimation	—	Cattle	1920 × 1080	2134	N	GitHub [262]	[216]
Tracking	Animal Tracking	Pig	2688 × 1520	2000	Y	PSRG [263]	[77]
			2688 × 1520	135,000	Y		[42]

**Note:** “—” indicates missing information; “Y” indicates a specific dataset has annotation; “N” indicates a specific dataset does not have annotation, and PSRG indicates Perceptual Systems Research Group. There can be more than one sizes of images, but we only listed one in the table because of presentation convenience.

## Data Availability

Not applicable.
